# Identifying Areas of Overlap and Distinction in Early Lexical Profiles of Children with Autism Spectrum Disorder, Late Talkers, and Typical Talkers

**DOI:** 10.1007/s10803-020-04772-1

**Published:** 2020-11-06

**Authors:** Eva Jiménez, Eileen Haebig, Thomas T. Hills

**Affiliations:** 1 Department of Psychology, University of Warwick, University Road, Coventry, CV4 7AL UK; 2 Department of Communication Sciences and Disorders, Louisiana State University, Baton Rouge, USA

**Keywords:** Autism spectrum disorder, Late talkers, Vocabulary, Semantic categories, Syntactic class

## Abstract

This study compares the lexical composition of 118 children with autism spectrum disorder (ASD) aged 12 to 84 months with 4626 vocabulary-matched typically developing toddlers with and without language delay, aged 8 to 30 months. Children with ASD and late talkers showed a weaker noun bias. Additionally, differences were identified in the proportion of nouns and verbs, and in the semantic categories of animals, toys, household items and vehicles. Most differences appear to reflect the extent of the age differences between the groups. However, children with ASD produced fewer high-social verbs than typical talkers and late talkers, a difference that might be associated with ASD features. In sum, our findings identified areas of overlap and distinction across the developing lexical profiles.

**Electronic supplementary material:**

The online version of this article (10.1007/s10803-020-04772-1) contains supplementary material, which is available to authorized users.

## Introduction

Children with autism spectrum disorder (ASD) have significant delays in early language acquisition (Charman et al. 2003; Ellis Weismer et al. 2011; Mitchell et al. 2006), but unlike late talking children, these language delays are accompanied by restricted interests, repetitive behaviors and a social communication deficit (American Psychiatric Association 2013). Might the language delay and the core deficits of ASD be related? This question highlights one of the central theoretical controversies within the ASD literature. That is, are the language delays associated with ASD merely adjustments along a continuum of development, where differences are primarily quantitative and along a single dimension (*the dimensional account*)? Or are the delays associated with ASD the result of a categorical difference in the way children with ASD learn language, giving rise to distinct language profiles that are not simply delayed versions of typical development (*the categorical account*)? Similarly, are the language profiles of children with ASD similar to late talking toddlers, or do they represent a unique profile unto themselves?

Although the current diagnostic criteria for ASD does not include lexical or grammatical language deficits (American Psychiatric Association 2013), receptive and expressive language delays have been found to differentiate children who will and will not go on to receive a diagnosis of ASD at ages as young as 12 months (Lazenby et al. 2016). Given this, previous research has examined the relation between various language domains and the language deficits in children with ASD (for an excellent review, see Eigsti et al. 2011). Though previous work has looked at early developmental patterns of the lexicon among children with ASD (Charman et al. 2003; Luyster et al. 2007; Rescorla and Safyer 2013; Ellis Weismer et al. 2011), the evidence needed to resolve the dimensional versus categorical account has been insufficient. The current study aims to address this problem by conducting an in-depth examination of the lexical composition of a large sample of children with ASD and to directly compare this with a large sample of children with typical language development as well as late talkers. Before we go on to describe our approach, we first describe the research supporting the dimensional and categorical accounts, lexical development in children with ASD and late talkers, and finally the putative role of social information in lexical development among children with ASD.

### The Dimensional and the Categorical Account of Language Development

In the dimensional account of language development (Gernsbacher et al. 2005; Rescorla 2009), children are placed along a continuum of language abilities, ranging from those with the poorest language skills to those with advanced language skills. Hence, the differences between a late talker and a typical talker are framed as being only quantitative (i.e., differences in the number of words produced), not qualitative (i.e., the type of words they produce). This account also implies that when late talkers and typical talkers are matched by language abilities (i.e., same number of words) the composition of their lexicons should remain similar. In contrast to the dimensional account, the categorical perspective of language development suggests that groups with language impairments demonstrate defining features of language development that do not align with characteristics of typical language development (Dollaghan 2004). In order to provide evidence for the categorical account, the identification of qualitative differences in the lexical profiles is useful because it can indicate the existence of potential atypical learning mechanisms. In this way, confirmation of lexical differences serves as a guidance for future investigations of cognitive processes, providing further insight into potential categorical differences.

To date, many studies have provided evidence suggesting that children with language delay and typically developing children show similarities in their patterns of language development (e.g., Ellis Weismer 2007; Rescorla 2009). The same has been proposed for children with ASD with regards to the proportion of syntactic and semantic classes (Charman et al. 2003; Luyster et al. 2007; Rescorla and Safyer 2013; Ellis Weismer et al. 2011). For instance, Charman et al. (2003) compared the proportion of words produced within syntactic classes (nouns, predicates, and closed-class words) in 87 preschool children with ASD to the normative sample for the MacArthur-Bates Communicative Development Inventory (CDI, Fenson et al. 1993). Charman et al. observed that the representation of the three syntactic classes across different vocabulary groups in the children with ASD was analogous to the pattern expected in a typical population. The proportion of semantic categories was also inspected in their sample. Children with ASD were reported to produce fewer words of the categories of ‘Sound Effects’, ‘Animals’, and ‘Toys’; however, none of these differences were greater than 20% different relative to the CDI normative sample. In a later study conducted by Luyster et al. (2007), the percentage of syntactic classes was similar to that of typically developing children, even after controlling for verbal and nonverbal mental age, confirming the descriptive findings of Charman et al. (2003).

Rescorla and Safyer (2013) investigated the syntactic and semantic composition of early vocabularies of children with ASD by employing a different vocabulary inventory, the Language Development Survey (LDS, Rescorla 1989). In their research, 45 children with ASD and 273 typically developing children were arranged into two overlapping groups by their total vocabulary: 1 to 49 words produced, and 1 to 310 words produced. Children with ASD and typically developing children who produced between 1 and 49 words had similar lexicons, for both syntactic and semantic classes. When examining the lexicons of the children who produced between 1 and 310 words, differences were found in the number of words produced in semantic categories; however, the differences appeared to be explained by the overall lower vocabulary skills in the children with ASD relative to the normative comparison sample. Across the quantitative and qualitative analyses that Rescorla and Safyer (2013) conducted, many similarities were observed between the children with ASD and typically developing children, which suggested that the sample of children with ASD demonstrated a significant delay instead of deviance in lexical development.

The significant delay in lexical development in children with ASD frequently challenges researchers when attempting to control for age differences when comparing children with ASD with children. Although previous work has documented that adults typically adapt their language input to the child’s language level (e.g., Dykstra et al. 2012; Hani et al. 2013; Paul and Elwood 1991), it is probable that older children are exposed to a somewhat different range of words which reflects changes in their immediate environment (e.g., "potty" instead of "diaper"). For this reason, an alternative comparison group to children with ASD is late talking children, who are closer in age. Although the majority of late talkers make significant language gains during the first years of life, many of them will experience persistent difficulties with some specific language abilities, such as in understanding and producing complex sentences at age five (Rescorla and Turner 2015) and in non-word repetition tasks (Conti-Ramsden et al. 2001). Predicting future outcomes and vocabulary structure in late talkers have been the subject of much investigation (for a review, see Hawa and Spanoudis 2014). For instance, Beckage and colleagues found that the structure of late talkers’ vocabularies have less semantic clustering and are less tightly connected than vocabulary-matched typical talkers (Beckage et al. 2011). Further, the emergence of word-learning biases has been computationally modeled in typical and late talkers’ vocabularies to confirm the difference in the lexical structure of these two groups, such as a difference in the reliance on the shape bias (Colunga and Sims 2017).

With regards to lexical composition, the percentage of the different syntactic and semantic categories in late talkers’ vocabularies have been found to be similar to vocabulary-matched typically developing children, with the exception of the percentage of nouns, which have been found to be lower (MacRoy-Higgins et al. 2016). Ellis Weismer et al. (2011) compared 40 toddlers with ASD and 40 late talkers, who were matched on expressive vocabulary. The authors found no differences between the two diagnostic groups across the 18 semantic categories on the CDI. Noun proportions were not examined in the sample; therefore, the question of whether the early vocabulary of children with ASD shows similar proportions of nouns to their late talking peers remained unanswered.

To date, a few studies on lexical composition give some weak support for the categorical account. Recent research has focused on individual lexical items within young children with ASD. In a large-scale study (209 toddlers with ASD and 272 typically developing toddlers), Bruckner et al. (2007) observed that 25 words in the CDI are more likely to be learned by children with ASD (i.e., had a large bias). Bruckner et al. suggested that ASD symptomatology, such as restricted object use, deficits in orienting to social cues, and social communication deficits, might be related to vocabulary differences between children with ASD and typically developing children. A more recent study by Lazenby et al. (2016) also showed that certain words on the CDI were statistically more or less frequent in the vocabularies of infants who later were diagnosed with ASD, compared to typically developing infants.

Despite the insubstantial evidence gathered to support the categorical view of language delay, findings that identify different learning biases in children with ASD warrant the continued examination of evidence for the dimensional or categorical account of language development (e.g., Field et al. 2016; Happé and Booth 2008; Pierce et al. 2011). Additionally, previous results from research that solely focused on the acquisition of nouns and verbs motivate us to further examine these two syntactic categories. For example, many studies have focused on a special case of lexical composition: the noun bias (e.g., Gentner 1982). The greater percentage of nouns in early vocabularies not only has been observed in typically developing toddlers, but also in 2- to 3-year-olds with ASD (Swensen et al. 2007). The noun bias has been linked to the well-known ‘naming explosion’ or spurt (Nelson 1973; Benedict 1979; Rescorla 1980; Goldfield and Reznick 1990). Many late talkers exhibited a reduced spurt, which suggests a potential link between noun acquisition and language delay (Rescorla et al. 2000). Different degrees of noun bias can be found in different languages, with the structure of the language being more influential in defining the intensity of noun bias than the parent linguistic input (Dhillon 2010). However, to our knowledge, previous research has not examined the possibility of identifying different degrees of noun bias and its relation to language abilities and ASD characteristics. The examination of the strength of noun bias seems relevant since previous studies have documented a weak or absent shape bias in children with ASD and late talkers, an important learning strategy for early noun learning (Jones 2003; Tek et al. 2008). In the present study, we will revisit the noun bias in the early vocabularies of children with ASD and late talkers with the aim to examine the strength of noun bias in these populations.

Although nouns are often the only syntactic class investigated in word learning studies, verbs have recently become the subject of interest among some researchers. Early verb acquisition may have a more important role in the later acquisition of grammatical abilities than nouns (Hadley et al. 2016). Some studies have focused on the type of verbs children acquire, which were classified according to syntactic features (transitive, intransitive and ditransitive; Olswang et al. 1997; Horvath et al. 2019) and to semantic features (manner and result verbs, punctual and durative verbs, number of event participants associated with its referent; Horvath et al. 2018b; Horvath et al. 2019). Late talkers who showed less change in MLU during a 9-week period produced fewer intransitive and ditransitive verbs than late talkers that showed greater MLU change (Olswang et al. 1997). Further, late talkers produced fewer manner verbs than their age-matched typical peers (Horvath et al. 2019). Regarding children with ASD, the syntactic bootstrapping strategies used to learn novel verbs by this population follow typical patterns (Shulman and Guberman 2007; Naigles et al. 2011; Horvath et al. 2018a, b). To our knowledge, the only other type of verbs investigated in children with ASD has been those that reflect mental states, which are described in the next section.

### Social Interest Deficit and Word Acquisition

Deficits in social orienting among young children with ASD have been widely reported, including aspects such as responding less to their names or making less eye-contact (Osterling et al. 2002). Additionally, Pierce and colleagues showed that 14-month-old infants with ASD attended to moving geometric shapes longer than to children performing actions (Pierce et al. 2011). Children with ASD also have been found to show a higher preference for verbal and non-verbal noise over clear adult speech (Klin 1991; Ceponiene et al. 2003). Different theories have suggested that this social disinterest in individuals with ASD either as a consequence of their deficits in social cognition (Social Cognitive Theory) or as a cause of their deficits in social cognition due to the diminished exposure to social situations (Theory of Social Motivation; for a discussion contrasting both theories see Chevallier et al. 2012).

Studies have examined the potential ways in which social communication deficits and difficulties in understanding the social world influence word learning in children with ASD. Difficulties with understanding social intentions have been found to negatively influence the acquisition of verbs and prepositions (Parish-Morris 2011). The acquisition of mental state verbs has been assessed (e.g., think, know, pretend) and suggested to be linked to weaknesses in Theory of Mind (Tager-Flusberg 1992). Tager-Flusberg examined language samples from children with ASD and children with Down syndrome and found that children with ASD produced fewer mental state verbs. Ziatas et al. (1998) found that older children with ASD had poorer comprehension of mental state verbs than verbal-mental-age-matched children with Asperger syndrome, typically developing children, and children with language impairment.

Horvath et al. (2018b) designed a word feature, where verbs where linked to the number of participants that are usually associated with them. Horvath and colleagues found that typically developing toddlers are more likely to produce verbs that can describe scenes that involve fewer events participants than those that label scenes with more participants. The authors argued that verbs with fewer participants are easier to learn because the syntax in which are embedded are easier to process. This word feature might be related to the degree of ‘socialness’ that children can perceive or be attracted to. In the current study, we explored this idea of words carrying social meaning. Verbs not only imply the number of event participants, but also the type of social interactions; for example, “smile” might evoke in the listener the act of someone smiling at someone else, or “share” might evoke someone sharing an object as part of a social interaction. Horvath et al. (2018b) demonstrated that typically developing children have greater difficulties in learning verbs that are associated with several event participants, one metric of the degree of socialness of the word. As such, given that children with ASD have difficulties attending to social cues, we wonder whether they would demonstrate pronounced challenges with learning highly-social words, relative to children who do not have ASD.

### Current Study

Our main aim in the present study is to contribute to the dimensional-categorical debate by disentangling the differences in the lexical composition that are related to language delay from those related to the ASD characteristics. We conducted a large-scale comparison of the early lexical profiles of children with ASD with that of typical talkers (TTs) and late talkers (LTs) to answer the following research questions:

1. Do children with ASD and LTs show a noun bias to a similar extent as TTs do? To answer this question, we compare the relative difference between the proportion of verbs and the proportion nouns between the talker groups. We hypothesized that the LTs and children with ASD may demonstrate a weaker noun bias, given that previous findings reported that these children do not demonstrate a shape bias (Jones 2003; Tek et al. 2008).
2. Do children with ASD, TT toddlers, and LT toddlers differ in the proportion of syntactic categories within their expressive lexicons? To test for differences, we grouped children by vocabulary size, as has been similarly done by Rescorla and Safyer (2013), and Charman et al. (2003), and deferred to a fairly conservative statistical test corrected for multiple tests using Bonferroni alpha corrections to determine significance (see Analysis Plan section for details). In the case where differences exist in the proportion of syntactic categories, we also asked whether these differences were age-related. We examine the make-up of the differences to determine whether these differences are a result of the extent of language delay (in relation to age). Additionally, we identified the words that can be potentially affected by normal developmental changes in early childhood and then drew potential links between these words and the categories/classes where the differences were found. We tentatively predicted that TT children may produce more nouns relative to the other groups because of a robust shape bias.
3. Do children with ASD show differences in the proportion of semantic categories compared to vocabulary-matched TTs and LTs? We followed a similar approach to addressing our first research question and provide a more detailed analysis description in the Analysis Plan section. Like in our syntactic analyses, we also asked whether semantic differences were associated to the age differences across our groups. We predicted that the majority of the semantic categories would be similar across the groups; however, if differences did appear, they would likely align with those identified by Charman et al. (2003; i.e., sound effects, animals, toys).
4. Do children with ASD produce verbs with less social features than children without ASD? We collected a set of social word norms to directly evaluate the potential influence of social context in driving early verb learning differences among children with ASD and children who do not have an ASD diagnosis (i.e., TTs and LTs). Our concentration on verbs allows us to extend recent work by Horvath et al. (2018b) on semantic (social) features that were initially used to examine early verb learning in typically developing toddlers. Unlike Horvath and colleagues’ measure of the number of participants associated to events and their associated actions, our social features rating has the advantage to capture the degree to which verbs represent sociably acceptable behaviors, such as ‘love’ or ‘hug’, and those less socially accepted behaviors, such as ‘hit’ or ‘hate’. We hypothesized that children with ASD would be less likely to be reported to produce verbs that are highly social.

## Methods

### Participants

We examined early expressive vocabularies of 118 children with ASD from word-level data collected using the CDI, obtained from the National Database for Autism Research in January of 2019 (NDAR; Payakachat et al. 2016). A comparison group of 4688 typically developing children with CDI data was downloaded from a public repository, Wordbank (Frank et al. 2017) in September of 2018. We compared our ASD sample against late talkers (LT) and typical talkers (TT; see Table 1 for participant characteristics) (Table 1). Late talking children were identified as those who fell at the 10^th^ percentile or below on the CDI norms. This threshold has been used previously in relevant studies on LTs (e.g., Ellis Weismer et al. 2011; D’Odorico et al. 2007; Moyle et al. 2007; Rescorla 2009). The maximum number of words produced by LTs in our sample was 250. Therefore, our sample of 118 children with ASD was selected following the criteria that none of them had more than 250 words in their productive vocabularies. Even after this restriction, the LT sample had lower vocabulary sizes than the TT group (LT: *M* = 43.1, TT: *M* = 72.7; *W* = 674,068, *p* < 0.001, *d* = 0.35, ${U}_{1}$= 24^1^) and the ASD group (*M* = 74.9; *W* = 35,030, *p* < 0.001, *d* = 0.32, ${U}_{1}$= 23). However, ASD and TT children had similar expressive vocabulary sizes (*W* = 230,755, *p* = 0.30). In our analyses, we addressed this difference in expressive vocabulary size by subgrouping children according to the total number of words produced. In addition to vocabulary size, the ASD, LT, and TT children differed in age, with the ASD group being the oldest group, followed by the LT group, and finally the TT group (age in months, ASD: *M* = 38.1, LT: *M* = 21.8, TT: *M* = 17.0; ASD vs LT: *W* = 50,419, *p* < 0.001, *d* = 1.22, ${U}_{1}$= 63; ASD vs TT: *W* = 468,383, *p* < 0.001, *d* = 0.54, ${U}_{1}$= 35; TT vs LT: *W* = 1,592,095, *p* < 0.001, *d* = 0.66, ${U}_{1}$= 41).

**Table 1:** Participant characteristics

Diagnostic Group	Number of children	Productive vocabulary size average, range^1^, (SD)	Age average^2^, range, (SD)	Checklist used
ASD	118	74.9 *1–250* (75.7)	38.1 *12–84* (15.9)	W&G = 31 (26.3%) W&S = 87 (73.7%)
TT	4142	72.7 *1–250* (66.3)	17.0 *8–29* (3.4)	W&G = 1739 (42%) W&S = 2403(58%)
LT	484	43.1 *1–248* (51.1)	21.8 *16–30* (4.6)	W&G = 28(50.8%) W&S = 456(94.2%)

*TDC* typically developing children, *TT* typical talkers, *LT* late talkers, *ASD* autism spectrum disorder. CDI forms: *W&G* words and gestures form, *W&S* words and sentences form

^1^Range of values are in italics. ^2^Age is in months

Data in our ASD sample were collected by different projects. The child data and study data for the children with ASD who were included in the current study can be inspected in NDAR, by searching our NDAR study https://doi.org/10.15154/1518553. All 118 children were diagnosed with ASD by employing either Autism Diagnostic Observation Schedule (*n* = 61; ADOS, Lord et al. 1999), ADOS-2 (*n* = 10; Lord et al. 2012), Childhood Autism Rating Scale (*n* = 14; CARS, Schopler et al. 1988), Diagnostic Statistical Manual (*n* = 33; APA 2013), or Autism Diagnostic Interview-Revised (*n* = 1; ADI-R, Rutter et al. 2003—though several children received the ADOS and the ADI-R). Visual Reception and Fine Motor subscales of the Mullen Scales of Early Learning (MSEL; Mullen 1995) have been widely used to assess non-verbal IQ. Given the extent of developmental delays in many children with ASD, age-equivalent scores are frequently reported (Bishop et al. 2011, 2015; Clark et al. 2017). Out of our sample, 98 children had a score for the Fine Motor subscale, with an age equivalent average of 26.8 months, ranging between 13 and 68 months; and 97 children had a score for the Visual Reception subscale, with an age equivalent average of 27.1 months, ranging between 11 and 54 months. The age equivalent of the ASD group (*M* = 26.9, SD 7.03) is still significantly higher than the chronological age of LT group (*W* = 34,134, *p* < 0.001, *d* = 0.6, ${U}_{1}$= 38), and TT group (*W* = 372,424, *p* < 0.001, *d* = 0.4, ${U}_{1}$= 27; age equivalent is an average of the two subscales; the 21 children without MSEL scores were excluded from this group comparisons).

### Syntactic Classes and Semantic Categories

The vocabularies of the children in the present study were assessed using two versions of the CDI: the CDI—words and gestures, normed on children between 8 and 18 months, and the CDI—words and sentences, normed on children between 16 and 30 months. To compare the early vocabularies of our groups of children, we conducted separate analyses according to semantic categories and syntactic classes.

For the syntactic analysis, we examine the two main types of words: nouns and verbs. Our motivations to study these two syntactic groups are based on the relevance that previous research established between their early acquisition and later language abilities (Benedict 1979; Hadley et al. 2016; Goldfield and Reznick 1990; Nelson 1973; Rescorla 1980; Rescorla et al. 2000). Nouns consisted of the words that were contained in the following CDI categories: animals (43 words), vehicles (14), toys (18), food and drink (68), clothing (28), body parts (27), furniture and rooms (33), and small household items (50). As in Bates et al.’s (1994) classification for the syntactic class of nouns, we excluded the following categories because it has been suggested they do not follow the typical growth of ‘true nominals’ (Snyder et al. 1981; Bates et al. 1994): sound effects and animal sounds (12), outside things (31), places to go (22), people (29), games and routines (25). The verb class included words classified as action words (103).

For the semantic analysis, all the CDI categories were considered. These are, in addition to the CDI categories mentioned so far: descriptive words (63), pronouns (25), questions words (7), prepositions and locations (24), quantifiers and articles (17), words about time (12), connecting words (6) and helping verbs (21). The items “inside/in” from the CDI Words and Sentences and “in” and “inside” from the CDI Words and Gestures were not used because when the item “inside/in” was marked it was not clear enough to confirm whether the child said one or two words. We decided to analyze those semantic categories excluded in our syntactic analysis for two reasons. Firstly, a considerable proportion of words in early vocabularies are composed of words from these semantic categories. Although these words have been suggested to not follow a typical nominal growth, we believe that any word from these categories could potentially be subject to an age effect, a factor of interest in the current study. Secondly, since we are building on previous studies that examined all the CDI categories (Charman et al. 2003; Luyster et al. 2007), we sought to analyze the same categories, which were comprised of the same word types, to contrast our results with those of these studies.

For each child, we computed the proportion of words produced for each syntactic class and each semantic category given the child’s total expressive vocabulary size. To calculate the expressive vocabulary size, we considered all words reported to be produced on the CDI. Then we sub-grouped the samples into bins according to the total number of CDI words produced. This approach allowed us to examine whether different patterns arise across different points of vocabulary development. This approach was particularly important because Bates et al. (1994) have suggested that vocabulary sizes between 1 and 25 or even 50 words are unstable. Furthermore, sub-grouping the samples into bins enabled us to control for differences in vocabulary size in our LT group. Therefore, for up to 100 words we use bins covering a range of 25 words. Between 101 and 250 words, we use bins covering a range of 50 words.

### Words Influenced by Developmental Stages in Early Childhood

To identify those words that are potentially associated to specific developmental stages throughout early childhood, we first split the word-level data into two age groups: the TTs as the ‘younger’ group, and the LTs and children with ASD as the ‘older’ group. Then, for each vocabulary bin, we computed the proportion of younger children and older children that produced each item/word separately for each group. Next, we subtracted the word proportions of the younger children with that of the older children per vocabulary bin. These subtractions resulted in positive numbers, which identify those words that younger children produced more often than older children, and negative numbers, which identify those words that older children produced more often than younger children. Finally, we extracted the top-10 most negative words and top-10 most positive words for each of the eight vocabulary bin comparisons.

We conducted a post-hoc examination of the words identified following the procedure just described, focusing only on those that belong to the categories where differences between LTs, TTs and children with ASD were previously found in our semantic analysis. Our objective is to determine whether the proportion of these semantic categories are related or not to developmental differences between the groups. To do this, we examined some word features associated to developmental changes that occur in early childhood. We concentrated on physical development as these changes are likely to influence the presence or absence of objects in the child’s environment, as well as the relation that children have with the objects. We expect to identify words like "diaper" and "bottle" in younger children, and words like "potty" and "fork" in older children. In the case of verbs, it is particularly challenging to infer how changes in physical development can influence verb acquisition as young children are able to learn the meaning of actions by observing other people (Huttenlocher et al. 1983). In addition, verbs can be learned as events and not actions, for example "walk" can be understood as the event of going to the park instead of the act of walking. However, we focused on the social aspect embedded in verbs, which we describe in the following section.

### Social Features in Verbs

In order to examine whether features associated with the core deficits of children with ASD influence early vocabulary development, we examined the social features of the words listed under the CDI Words and Sentences Action Words category. Social ratings for each verb were collected from a sample of 54 adults using a survey that was distributed on Amazon’s Mechanical Turk platform. The participants lived in the United States of America and self-reported to be native English speakers. Thirty-one participants identified as male, 21 identified as female, and two participants identified as “other”. The average age of the participants was 35.9 years (range 22–72 years) and the average reported household income was $47,444 (range $7000–$120,000). The sample was 83.3% White, but also included four Asian individuals, two Black individuals, and one “other”. Additionally, four individuals reported to be Hispanic.

The participants were given the following prompt “For each of the following words, please type in a number between 1 and 10 to rate how social each word is (1 = not social, 10 = extremely social). A word is more social if it typically involves interacting with other people. A word is less social if it typically does not involve other people.” This approach to measuring social features of the verbs was similar to the approach used by Horvath et al. (2018b). The order of the words was pseudo-randomized so that it was not in alphabetical order. Three items were added to the survey to test for attention by asking the participant to select a specified word from a list of three words. Every participant passed these items; therefore, no participants were excluded. The average social rating for each verb was calculated. Then, we calculated the median social rating score for the verbs reported to be produced by each child in our sample. Following this, to control for the higher proportion of verbs that our sample of children with ASD produced, we subdivided our sample according to vocabulary bins of verbs produced: 1 to 25 verbs, and 26 to 50 verbs. Not all children in our sample were included in this analysis since some children with small vocabularies produced no verbs. The subsample analyzed comprised of 83 children with ASD, 233 LTs, and 2457 TTs.

### Analysis Plan

We chose to conduct non-parametric pairwise comparisons using the Wilcoxon rank-sum test to test for group differences since the distribution of proportions across vocabulary sizes violated the assumption of homogeneity of variance. To be able to control for vocabulary size, we tested the children within each vocabulary size bin (see Table 1 for vocabulary sizes analyzed). The Benjamin and Hochberg false discovery rate procedure was implemented in each test performed. Additionally, all *p*-values were corrected using the Bonferroni method as we conducted several distinct analyses on the sample; corrected *p-*values are reported.

In order to explore the influence of age on word proportions, we considered any difference that emerged between the three groups in one or more vocabulary bins. Since many semantic categories are composed of a few words, there is a chance that differences between two groups emerge in some of them; therefore, to minimize type 1 error, we will only acknowledge differences between groups if we found significant differences in at least two vocabulary bins. We only report significant results in the main text of this manuscript; however, results from all the analyses can be found in Online Appendix A. To evaluate the effect size of the significant results, we report two statistics following the suggestion made by Fritz et al. (2012). The first statistic is the well-known Cohen’s *d*, which we interpret following the Cohen’s convention (1988). In addition, to facilitate the interpretation of the relation between the groups’ distributions, we also report ${U}_{1}$, also created by Cohen (1988), which is the percentage of non-overlap between the two distributions.

## Results

### Noun Bias

The LT and ASD groups showed a higher proportion of nouns (ASD: *Mdn* = 0.47; LT: *Mdn* = 0.37) than verbs (ASD: *Mdn* = 0.08; LT: *Mdn* = 0.007; ASD: *Z* = 8.4, *p* < 0.001; LT: *Z* = 18.52, *p* < 0.001). Typical talkers also showed a higher proportion of nouns (*Mdn* = 0.48) than verbs (*Mdn* = 0.04; *Z* = 8.21, *p* < 0.001). The effect size of all noun bias analyses were very large (ASD: *d* = 2.0, ${U}_{1}$= 81; LT: *d* = 2.70, ${U}_{1}$= 90; TT: *d* = 3.58, ${U}_{1}$= 96). To further explore this noun bias, we subtracted the proportion of verbs from the proportion of nouns for each child in our sample. Then, we compared these verb-noun gaps between our three groups in a subsample of the children with vocabulary sizes between 1 and 75, which includes the vocabulary bins where nouns and verb differences were found in our syntactic analysis (reported in the next section; ASD: 77 children; LT: 400 children, TT: 2646 children). The ASD group and the LT group had the smallest verb-noun gaps (ASD: *Mdn* = 0.27; LT: *Mdn* = 0.30), and the TT group showed the largest gap (*Mdn* = 0.39). The ASD group differed from the TT group (*W* = 76,278, *p* < 0.001) but not from the LT group (*W* = 13,995, *p* > 0.05). There were significant differences between the TT group and the LT group (*W* = 413,471, *p* < 0.001). The effect sizes of the noun–verb gap analyses were generally small (ASD vs TT: *d* = 0.1, ${U}_{1}$= 8; LT vs TT:* d* = 0.26, ${U}_{1}$= 21).

### Syntactic Classes and Semantic Categories

**Fig. 1:**
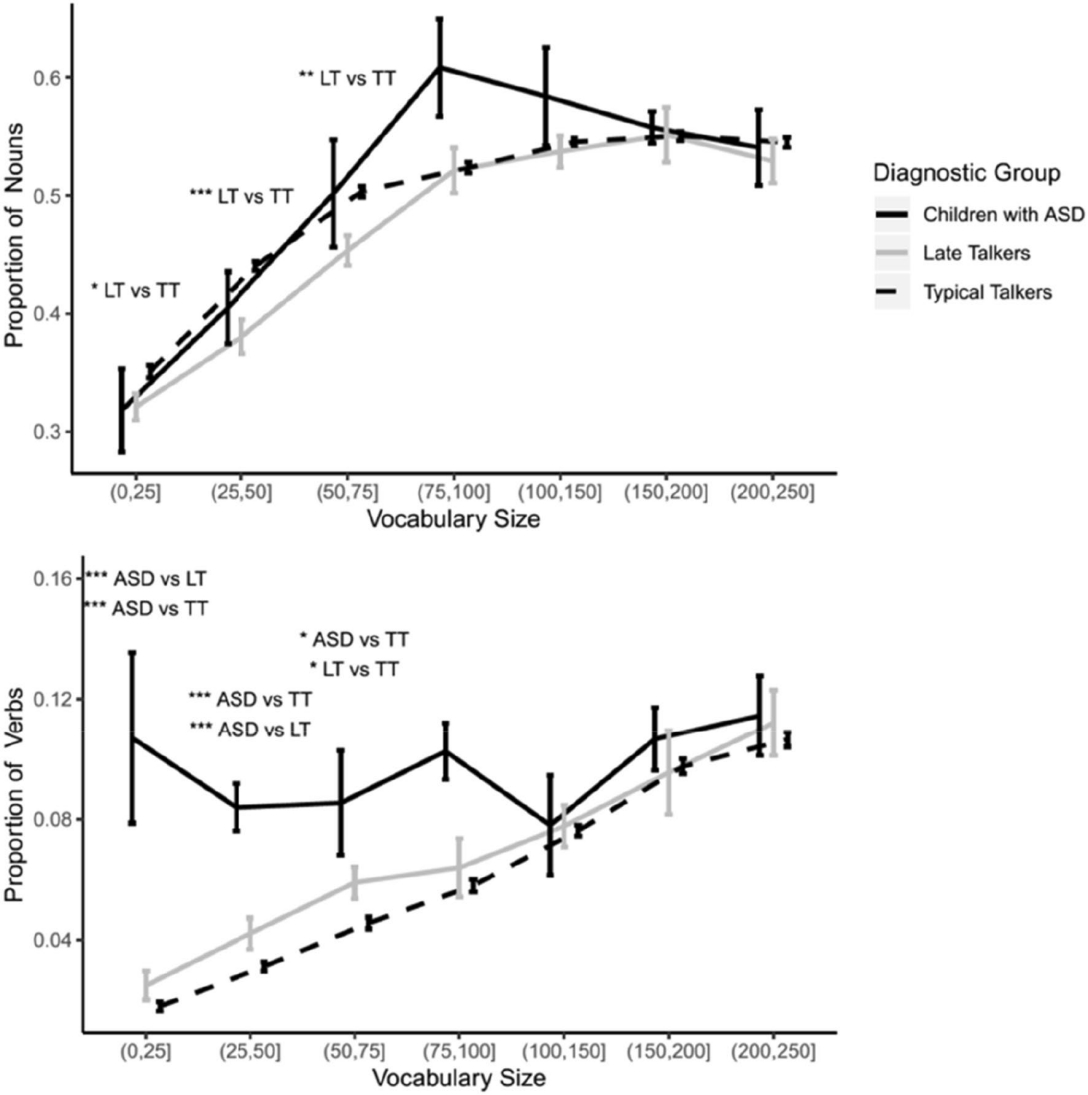
Proportion of syntactic classes in the vocabulary of children with ASD, late talkers, and typical talkers. Error bars, signifying standard error of the mean, have been shifted slightly to facilitate visibility. Asterisks indicates a significant group difference: *p < .05; **p < .01; ***p < .001

We first compared the early production of nouns and verbs across children with ASD, TTs, and LTs. Figure 1 depicts the proportion of words produced within each syntactic class according to vocabulary bin size for the ASD, TT, and LT groups. Our analysis on the proportion of nouns revealed that there were no differences between children with ASD and either LTs or TTs (see Table 2). Late talkers produced a lower proportion of nouns than TTs for vocabulary sizes 1 to 25, 26 to 50, and 51 to 75. The effect sizes of the noun analyses were generally small (*d* = [0.1*,*0.3]; ${U}_{1}$= [8*,* 21]). Verbs revealed a different pattern. Children with ASD produced a higher proportion of verbs than TTs at vocabulary bins 1 to 25, 26 to 50, and 51 to 75; and produced a higher proportion of verbs than LTs for the two smallest vocabulary size bins. Late talkers exhibited a larger proportion of verbs than TTs for the 51–75 vocabulary size bin. The effect sizes of the verb analyses ranges from small to medium for the ASD vs TT comparisons (*d* = [0.2,0.4]; ${U}_{1}$= [15, 27]), medium to large for the ASD vs LT comparisons (*d* = [0.4,0.9]; ${U}_{1}$= [27, 52]), and small for the TT vs LT comparison (*d* = 0.3; ${U}_{1}$= 21). Given that the Bates et al. (1994) syntactic classifications have been frequently used in previous research (e.g., Braginsky et al. 2019; Charman et al. 2003; Luyster et al. 2007; MacRoy-Higgins et al. 2016; Roy, Frank et al. 2015), we also classified the words following their approach. Bates et al. (1994) categorized CDI words as either nouns, predicates (adjectives and verbs), or closed class words. Analyses of these syntactic classes replicated the significant differences between the groups for the nouns and verbs/predicates and revealed no differences for close-class words (see Online Appendix B).

**Table 2:** Wilcoxon rank sum test for nouns and verbs

Syntactic class	Vocabulary size	Mean ASD	Mean LT	Mean TT	ASD vs TT			ASD vs LT			TT vs LT		
					*W*	*p*	*d*	*W*	*p*	*d*	*W*	*p*	*d*
Nouns	1–25 words	0.32	0.32	0.35	24,914.5	> .05	–	5707.5	> .05	–	193,148.5	< .05	0.1
	26–50 words	0.40	0.38	0.44	7179.0	> .05	–	834	> .05	–	42,368	< 0.001	0.3
	51–75 words	0.50	0.45	0.50	3005.0	> .05	–	413.5	> .05	–	17,023	< 0.01	0.3
Verbs	1–25 words	0.11	0.025	0.018	40,113.0	< .001	0.2	8092.5	< .001	0.4	169,001	> .05	–
	26–50 words	0.084	0.042	0.031	15,090.0	< .001	0.4	1221.5	< .001	0.9	28,761	> .05	–
	51–75 words	0.085	0.059	0.046	4167.5	< .05	0.2	423.5	> .05	-	10,004	< .05	0.3

Groups considered in the analysis are the ASD group, LT (Late talker) group, and TT (Typical talker) group. Only the three vocabulary bins that were significant are displayed. Full results for all vocabularies can be found in Online Appendix A, Table A1. All *p*-values were first corrected using the BH method then corrected again using the Bonferroni method (i.e., corrections accounted for comparisons for the three syntactic classes)

**Fig. 2:**
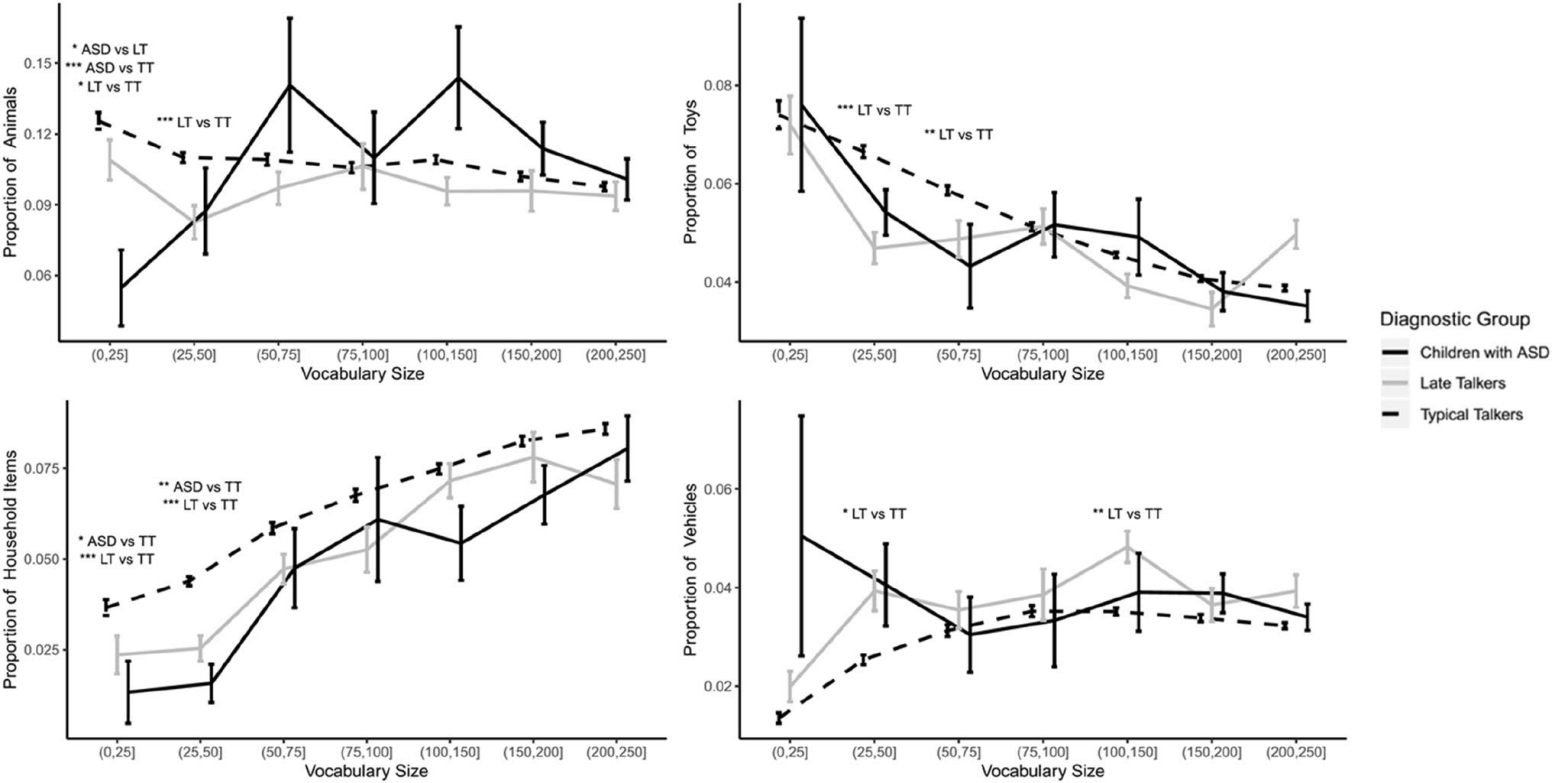
Proportion of Animals, Small Household items, Toys and Vehicles in the vocabulary of children with ASD, late talkers and typical talkers. Error bars, signifying standard error of the mean, have been shifted slightly to facilitate visibility. Asterisks indicates a significant group difference: *p < . 05; **p < . 01; ***p < . 001

In addition to examining the syntactic organization of early vocabulary development, we inspected the 22 semantic categories on the CDI. We found differences between the groups in the semantic categories of Action Words, Animals, Small Household items, Toys and Vehicles. We will not discuss Action Words since verbs were already discussed for the syntactic analysis. The following results are shown in Fig. 2 and Table 3. We found significant differences in the proportion of animal words between all groups at vocabulary sizes 1 to 25, where TTs presented the highest proportions, followed by LTs and then the children with ASD with the lowest proportion. In the vocabulary bin of 26 to 50 words, LTs and children with ASD produced a similar proportion of animal words, and only LTs significantly differed from the TTs, with TTs producing the highest proportions. In our small household items findings, LTs and children with ASD presented similar proportions at small vocabularies only. Both differed from the TTs who produced more of these words in vocabulary sizes 1 to 25 and 26 to 50 words. Our Toy words finding revealed that LTs and children with ASD had similar proportions of toy words, but only LTs differed from the TTs in vocabulary bins 26 to 50, and 51 to 75, with TTs showing the highest proportions. Similarly, LTs and children with ASD showed similar proportions of vehicle words, with LTs being the only of the two groups to differ from the TTs, who produced more vehicle words at vocabulary sizes 26 to 50, and 101 to 150 words. Overall, out of the seven vocabulary sizes analyzed, differences were found only in two vocabulary sizes in each semantic category that we discussed. These were mostly small vocabulary sizes and mostly between LTs and TTs. In addition, the differences found had a small effect size (ASD vs TT: *d* = [0.2, 0.3]; ${U}_{1}$= [15, 21]; LT vs TT:* d* = [0.2, 0.3]; ${U}_{1}$= [15, 21]), and one difference had medium effect (ASD vs LT:* d* = 0.4; ${U}_{1}$= 27).

**Table 3:** Wilcoxon rank sum test for differences across semantic categories

Semantic category	Vocabulary size	Mean ASD	Mean LT	Mean TT	ASD vs TT			ASD vs LT			TT vs LT		
					*W*	*p*	*d*	*W*	*p*	*d*	*W*	*p*	*d*
Animals	1–25 words	0.055	0.11	0.13	16,696	< .001	0.3	4309.5	< .05	0.4	194,996.5	< .05	0.2
	26–50 words	0.087	0.083	0.11	6392	> .05	–	784.5	> .05	–	43,113	< .001	0.3
Small Household Items	1–25 words	0.013	0.024	0.037	22,106.5	< .05	0.2	5697	> .05	–	204,735.5	< .001	0.2
	26–50 words	0.016	0.025	0.044	4594.5	< .01	0.3	649	> .05	–	44,014.5	< .001	0.3
Toys	26–50 words	0.054	0.046	0.067	6762	> .05	–	910	> .05	–	44,596.5	< .001	0.3
	51–75 words	0.043	0.049	0.059	1997	> .05	–	322	> .05	–	17,341	< .01	0.3
Vehicles	26–50 words	0.041	0.049	0.025	10,798.5	> .05	–	781.5	> .05	–	25,856	< .05	0.2
	101–150 words	0.039	0.048	0.035	3238.5	> .05	–	184.5	> .05	–	5333.5	< .01	0.3

Results obtained from post-hoc Wilcoxon rank sum tests for the four semantic categories that showed significant differences between the groups: Animals, Small Household Items, Toys and Vehicles. Groups compared in the analysis are the ASD group, LT (Late talker) group, and TT (Typical talker) group. Only the vocabulary bins that were significant are displayed. Full results for all vocabularies can be found in Online Appendix A, Table A2. All *p*-values were firstly corrected using the BH method then corrected again using the Bonferroni method (correcting for comparisons across 22 semantic categories)

In contrast to our syntactic analysis, our semantic analysis presents the disadvantage that many of the CDI categories are composed of a small number of words. Although the statistical tests detected significant differences in proportions between the groups, it is necessary to consider the relative size of the differences in terms of number of words that children usually produce at the corresponding vocabulary sizes. In our sample, 90% of children with a vocabulary size between 1 and 50 words produced between 0 and 2 small household items (1 to 25 words: *M* = 0.41, SD 0.70; 26 to 50 words: *M* = 1.57, SD 1.46), and between 0 and 5 animals (1 to 25 words: *M* = 1.51, SD 1.52; 26 to 50 words: *M* = 3.96, SD 2.41); 94% of the children with vocabulary sizes of 26 to 75 words produced between 0 and 4 toys (26 to 50 words: *M* = 2.35, SD 1.27; 51 to 75 words: *M* = 3.54, SD 1.36). Regarding vehicle words, 90% of children with vocabulary sizes of 26 to 150 words produced between 0 and 5 vehicles (26 to 50 words: *M* = 1.02, SD 1.16; 101 to 150 words: *M* = 4.50, SD 2.27).

### Words Associated with Developmental Stages

To examine the potential influence of age in our findings of lexical differences, we explored word-level differences. Table 4 shows the set of words whose production are potentially influenced by age in children at similar stages of vocabulary development. We only display and discuss those CDI categories where differences were identified in our semantic analysis; however, the full results can be found in Online Appendix C. The number of words that we identified reflect the proportions that the corresponding comparison groups showed in our semantic analysis; that is, more words were identified in the group that previously showed higher proportions in the respective CDI category.

**Table 4:** Words that older and younger children produce which are potentially related to differences in development

Animals		Small household items		Toys		Vehicles	
Younger children	Older children	Younger children	Older children	Younger children	Older children	Younger children	Older children
Bear (*c*, *e*)	Bee (*c*)	Bottle (*a*, *b*, *c*, *d*)	Plate (*f*)	Balloon (*a*, *b*, *c*)	Ball (*a*)	Airplane (c)	Bus (*e*)
Bird (*a*, *b*, *d*)	chicken (*g*)	broom (*f*)		Block (*e*)	Playdough (*g*)		Car (*a*, *b*)
Bunny (*b*)	Horse (*d*)	Keys (*d*)		Book (*a*, *b*)			Helicopter (*e*)
Cat (*b*)	Lion (*g*)	Spoon (*e*)		Doll (*e*)			Motorcycle (*e*)
Dog (*b*)	Penguin (*g*)			Toy (*f*)			Truck (*d*)
Duck (*b*)							
Kitty (*a*, *b*)							
Owl (*g*)							
Pig (*g*)							
Teddy bear (*c*)							

Letters in parenthesis represent the vocabulary size where the word was identified*. a* 1 to 25 words; *b* 26 to 50 words; *c* 51 to 75 words; *d* 76 to 100 words; *e* 101 to 150 words; *f* 151 to 200 words; *g* 201 to 250 words

We found age differences in words related to the physical readiness for potty training, and the development of the digestive system. Regarding small household items, two of the words that younger children are more likely to produce, "bottle" and "spoon", seem to be related to early stages of feeding. In our examination, we also identified other words that we expected to be affected by age but that belong to other semantic categories where group differences were not observed. Some examples are "bib" or "cracker" in the younger group, and "go potty" and "candy" in the older group.

We also explored features related to fine versus gross motor skills. These features can be applied to those words that represent objects that children are normally allowed to manipulate, i.e., toys. In this sense, we expect that older children would engage more often in playing with toys where fine motor skills are required. In the case of the toy-related words, which are composed of the CDI categories of Animals, Toys, and Vehicles, we identified words that require fine motor skills in both groups (older group: "play-dough"; Younger group: "block"). Interestingly, the older children are likely to produce toys with small rotating parts ("bus", "car", "helicopter", "motorcycle", "truck"), which contrast with the high proportion of toys produced by younger group that are generally characterized for the lack of small mobile pieces ("doll", "teddy bear", "balloon").

### Social Features and Verb Acquisition

Regarding the social ratings for verbs given by adults, the highest rating value was 9.2 and corresponded to the words “kiss” and “hug”; other highly rated words included “help” (8.3) and “tickle” (8.1). The lowest social rating value was 1.6 and corresponded to the word “rip”; other words that received low social ratings included “sweep” (1.8) and “jump” (2.0). The average social rating value was 3.9; words with similar scores are “see” (3.7), “cry” (4.0), and “cook” (4.2). Low socially acceptable actions that involve more than one participant, like “hit” and “hate”, received medium rating values (4.6 and 4.2 respectively). Apart from “kiss”, “hug” and “help”, other high socially desirable actions received high scorings, such as “share” (8.6) and “love” (8.5).

**Fig. 3:**
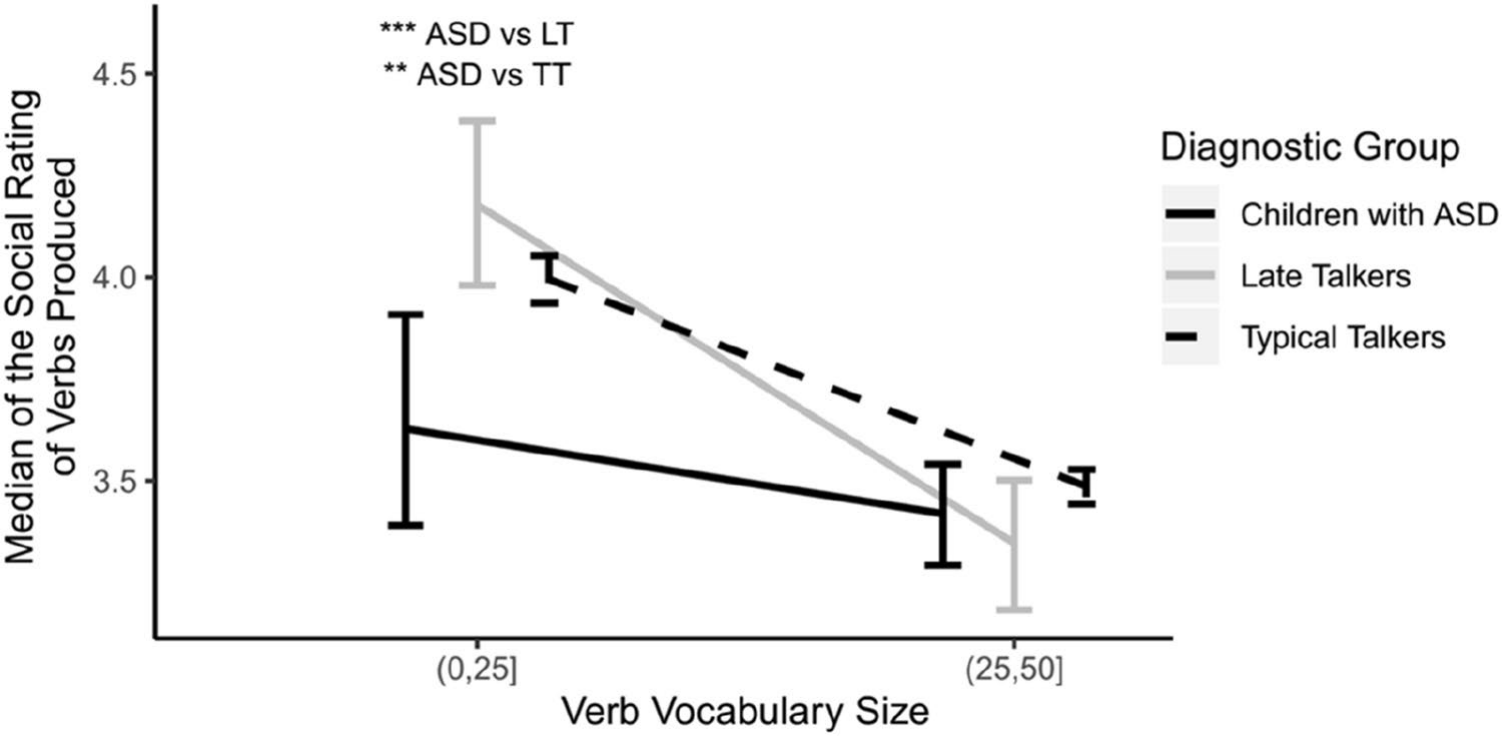
Median social rating scores for verbs that children with ASD, late talkers and typical talkers produced relative to the total number of verbs produced. Error bars, signifying standard error of the mean, have been shifted slightly to facilitate visibility. Asterisks indicates a significant group difference: **p < .01; ***p < .001

To address our last research question—whether characteristics associated with ASD symptomatology, specifically deficits in social abilities, relate to verb production—we examined the relationship between verb acquisition and the degree to which verbs are associated with social interactions. We compare children with the same number of verbs in their vocabularies. One group of children produced between 1 and 25 verbs (TT *n* = 2457, LT *n* = 233, ASD *n* = 83), and the other group children produced between 26 and 50 verbs (TT *n* = 162, LT *n* = 9, ASD *n* = 9). As can be observed in Fig. 3, children with ASD within the first 25 verb bin produce verbs with significantly lower social ratings (*Mdn* = 3.1) when compared to TTs (*Mdn* = 3.6, *W* = 80,239, *p* < 0.01) and to LTs (*Mdn* = 3.7, *W* = 6931, *p* < 0.001). No differences were found between the LTs and TTs (*W* = 264,746, *p* > 0.05). The effect size was small for the ASD vs TT comparison (*d* = 0.13; ${U}_{1}$= 10), and medium for the ASD vs LT comparison (*d* = 0.44; ${U}_{1}$= 30).

From the developmental point of view, Fig. 3 suggests that all groups generally produced more high-social verbs at the early stages of vocabulary development (i.e., at vocabulary sizes 1 to 25 words). However, only TTs showed significant differences between the two verb vocabulary bins (verb vocabulary size (0, 25] *Mdn* = 3.6, *M* = 4.0; verb vocabulary size (25,50] *Mdn* = 3.5, *M* = 3.5; *W* = 224,078, *p* < 0.01). This difference had a small effect size (*d* = 0.11; ${U}_{1}$= 9). LTs and children with ASD did not differ across the two vocabulary sizes analyzed (LTs: verb vocabulary size (0,25] *Mdn* = 3.7, *M* = 4.2; verb vocabulary size (25, 50] *Mdn* = 3.3, *M* = 3.5; *W* = 1388, *p* = 0.09; ASD: verb vocabulary size (0,25] *Mdn* = 3.1, *M* = 3.6; verb vocabulary size (25, 50] *Mdn* = 3.4, *M* = 3.4; *W* = 324.5, *p* > 0.05).

## Discussion

The current study identified group differences across syntactic classes, semantic categories, and social features of verbs. Our findings highlighted group differences that primarily occur at the earliest stages of lexical development. In what follows, we discuss our findings in the context of the broader literature.

### Noun Bias

The children with ASD showed a noun bias in their vocabularies, supporting previous findings (Swensen et al. 2007). However, in our attempt to measure the strength of the noun bias (proportion of verbs subtracted form proportion of nouns, or noun–verb gap) we found that LTs and children with ASD showed a weaker noun bias compared to TTs. In addition, LTs and children with ASD showed similar sizes of the noun–verb gap. Within the noun bias literature, it has been determined that the language spoken at home is the main factor that drives the strength of the noun bias (Dhillon 2010). Since all the children in our sample were English speakers, we can discount this effect and posit that late language onset is a factor that regulates the size of the noun–verb gap. To help interpret the noun–verb gap findings, it is helpful to consider the syntactic comparisons. We found medium to large effect sizes observed in our verb comparisons. Although all of the groups demonstrated a noun bias, the comparatively high proportion of verbs that the children with ASD produce mainly influences the size the verb-noun gap difference relative to the TTs. However, it is important to remember that the effect sizes of the noun–verb gap group differences were generally small; as such, these findings underscore the consistency of the noun bias across groups but identify interesting differences in its degree.

### Syntactic Classes and Semantic Categories

Within our comparison of the proportion of the syntactic classes, the verb differences were the most striking. The ASD group, which consisted of older children with the largest language delays showed the highest proportions of verbs, followed by the second oldest group (LTs) and finally the youngest group (TTs) with the lowest proportions. A possible explanation might be that, since LTs and children with ASD are older, their cognitive abilities are more mature than verbal-matched typical talkers, as demonstrated by the comparison between the ASD group’s MSEL age equivalence and chronological age of the TT and LT groups. Alternatively, the age differences also indicate that the older children likely experienced additional exposure to verbs. Future work is needed to determine the exact factors that drove the verb differences; the data analyzed in the current study are insufficient to identify the exact mechanisms that might cause these differences. With regards to nouns, LTs showed lower proportions than TTs in the early vocabulary sizes (1–75 words). This is consistent with findings from MacRoy-Higgins et al. (2016), who also found that late talkers had a lower percentage of nouns than age-matched and verbal-matched children. MacRoy-Higgins and colleagues suggested that the lower production of nouns in LTs might be an indication of a late-arriving vocabulary spurt. Interestingly, the proportion of nouns in children with ASD in our sample falls between the LTs and the TTs, something that cannot be explained by age since the ASD group has the largest language delay. This would mean in theory that the exact moment of the vocabulary spurt in children with ASD (relative to their vocabulary size) should be somewhere in between that of LTs and TTs (typically, a spurt has been observed once children acquire between 50 and 100 words; Bates et al. 1994). Nevertheless, the effect sizes for the noun differences are generally lower than those for the verb differences.

Differences were found in four CDI semantic categories: Small Household Items, Animals, Toys and Vehicles. With respect to small household item words, the acquisition of the word "bottle" by TTs could have had a sufficient impact on the detected proportion differences due to the generally low production of small household items (0 to 2 words) at small vocabularies. The fact that the two oldest groups, LTs and children with ASD, differed from the youngest group, TTs, supports the argument that the differences identified are likely to be related to developmental discrepancies between the groups (i.e., age).

Curiously, we found differences in three CDI categories that are related to play: animals (real or toy), vehicles (real or toy), and toys. Children typically produce a wider range of words that belong to these categories compared to Small Household Items (up to five words in the vocabulary sizes where differences were found). A noticeable difference between the type of toys that older children acquire compared to vocabulary-size-matched younger children is that they generally have features that can be manipulated where advanced fine motor skills are needed (e.g., vehicles, playdough). Parents of young toddlers might avoid giving their children toys with these characteristics which might represent a choking hazard, which potentially influence the type of words to be produced by the child. The differences in proportion of animals produced in our sample follows the same pattern of differences in age, which support the age effect hypothesis. However, this is not the case for toy words and vehicle words, where the production of these words by children with ASD are somehow placed between TTs and LTs. These findings might suggest an association between word acquisition and play skills. The level of functional play in 3- to 5-years-old children with ASD has been found to be less elaborate and less diverse than vocabulary- and developmental-matched typically developing children and children with Down syndrome (Williams et al. 2001). In a previous study, late talkers were also found to produced less sophisticated play, initiating fewer play scripts and producing more instances of non-functional play (Rescorla and Goossens 1992). Our difference in toy-related words in our ASD and LT groups fits well with the prior literature documenting connections between play skills and language (e.g., Conner et al. 2014; Ingersoll and Schreibman 2006). In any case, the effect sizes of these findings are small and suggest that any influence related to differences in development or to differences in play-skills are likely to have a weak impact on the semantic composition of the children’s vocabularies.

Our study introduces some distinct methodological aspects in comparison to previous studies that might explain our findings. First, we differ from these studies in that we included late talkers as a third comparison group, allowing us to consider the potential effect of age or language delay. Second, we use a different vocabulary grouping system. Charman et al. examined vocabulary bins of many different sizes, starting with groups with small differences in the number of words produced (e.g., 1 to 5 words), up to groups with very large differences among children (e.g., + 50 words). The aim of the Charman et al.’s (2003) arrangement was to facilitate the comparison of the CDI vocabularies of children with ASD with the normative sample collected by Bates et al. (1994), who also grouped children in this manner. Bates et al. (1994) claimed that early lexical development is characterized as being an ‘unstable period’. In the view of our results, it is perhaps the case that by grouping children in slightly larger vocabulary bins and one might be able to control for this predicted early variability (i.e., one bin of 1 to 25 words instead of three bins of 1 to 5, 6 to 10 and 11 to 20 words). Luyster et al. (2007) used no vocabulary grouping, and Rescorla and Safyer (2013) examined a group of children with very large vocabulary size differences (1 to 49 words, and 1 to 310 words). In addition to the methodological differences in vocabulary size bins, the two groups in the Ellis Weismer et al. (2011) sample (ASD vs LTs) differed in age. In comparison to our sample, the age gap between the ASD group and the LT group is much larger than that of Ellis Weismer et al.’s study (mean ASD: 30 months; mean LT: 25 months). Further, our ASD sample size nearly triples Ellis Weismer et al.’s ASD sample and our late talker sample size is twelve times larger than their late talker sample.

### Acquisition of High Social Verbs

Our analysis of social words found that children with ASD learned fewer highly social verbs than language-matched TTs and LTs with small verb vocabulary sizes. This finding may indicate that typically developing children may more reliably use social information to learn verbs. Previous research has suggested that verb learning is negatively influenced by a poor understanding of the speaker’s social intentions in children with ASD (Parish-Morris 2011). We contribute to this research by identifying social features not associated with the social interaction present at the moment of learning (i.e., adult speaking with the child), but in the words themselves, and that acquiring high-social verbs might be challenging to children who show difficulties in understanding social events. What seems to be contradictory is that, even though children with ASD showed a lower tendency to learn high social verbs, they managed to learn a higher proportion of verbs overall than their vocabulary-matched typically developing peers, suggesting that social features only have an influence on the type of verbs they acquire, not the quantity. This fact might be indicating an atypical use of verbs, which would be more directed to instrumental goals, rather than to social goals such as requesting for a joint attention activity or a coordinated and reciprocal play activity.

Since verbs associated with many event participants are harder to process by typically developing children (Horvath et al. 2018b), another possible explanation of why children with ASD produce less high social verbs might be related to the difficulties they face with complex syntax, a characteristic typically observed in ASD (Tager-Flusberg and Joseph 2003). Conversely, the number of social features in the first verbs learned by LTs resemble that of TTs, indicating that LTs are equally likely to attend to and learn verbs that are typically associated with interactions with other people. Late talking toddlers have been previously found to socialize less than typically talking toddlers (Irwin et al. 2002); however, our findings suggest that this may not negatively impact their learning of social verbs.

We also found that TTs with small verb vocabularies had more high-social verbs on average than TTs with larger vocabularies, indicating a preference for producing high-social verbs earlier. This is suggestive of a general social-word bias in early word acquisition, a word learning preference that, to our knowledge, no study has reported before. Although the visualization of our results suggests that LTs and children with ASD showed this social-word bias, our analysis determined that there were not significant differences between the two vocabulary sizes. The number of LTs and children with ASD in the large vocabulary groups were quite small, which provides us with low power to detect small effect sizes. Therefore, although children with ASD showed a reduced acquisition of high-social verbs at small vocabularies, we cannot discard the possibility that children with ASD have a weak social-word bias.

### Limitations and Future Research

Although the current study has several strengths that allowed for novel insights into early lexical development in children with ASD, there are a number of limitations that must be kept in mind. The first limitation relates to the lack of information about the composition of the samples. Although we were able to gather information about ASD evaluation protocols used and nonverbal cognitive skills from the majority of the children with ASD, we were not able to gather this information for the children in the TT and LT groups. This means that there is a possibility of having cases of children in our TT and LT groups who could potentially be identified as having ASD at later times. This risk is especially relevant for the LT group. However, it should be noted that the incidence of late talkers is higher than that of ASD. The second limitation is the criteria chosen to create semantic categories. We used the categories given by the CDI and treated the words within each category as semantically similar. Although this mirrored method used in the prior literature, some special cases like sound effects could be categorized differently. Third, and most notably, we do not have data that provide sufficient insight into *why* lexical differences exist between the groups. Future experimental studies are needed to provide the necessary mechanistic accounts that explain the areas of distinction in the lexical profiles demonstrated by late talkers and children with ASD.

Future research should further investigate children’s learning of verbs with varying social features in controlled learning situations to confirm our suggested interpretation related to children with ASD learning fewer high social verbs. Specifically, it would be of interest to determine whether the potential lower acquisition of high social verbs is related to the number of participants involved in the action or whether it is related to the degree of the social interest in the action. We failed to prove whether LTs and children with ASD show a social-word bias due to the small size of one of the comparison groups. Future research could confirm whether these children have a social-word bias at all by repeating the analysis with a larger sample of children with large verb vocabularies. In addition, our finding of the reduced noun-verbs gaps in children with ASD and LTs motivate future research to further investigate the relation between this gap and language delay. Finally, based on our semantic category findings, future work should examine the relationship between specific play skills and word acquisition.

## Conclusion

Although the proportion of words in the vocabularies of children with ASD is similar to typically developing vocabulary-matched children in most semantic categories (supporting the dimensional account), the current study is the first to directly compare these three groups and to identify differences in two syntactic classes and four semantic categories. Most of the differences were found at small vocabularies and with small or medium impact on the composition of the children’s vocabularies. In addition, the pattern of the group differences suggest age as a factor that drives most of the differences. In addition to identifying similarities in many semantic categories, we also documented that LTs, TTs, and children with ASD demonstrate a noun bias; however, the degree of the noun–verb gap differed between the groups. We found that LTs and children with ASD had a smaller noun–verb gap relative to TTs, suggesting a link between language delay and noun–verb acquisition. Further, our results suggest that verb acquisition in children with ASD is influenced by the social features embedded in verbs, with these children primarily acquiring less-social verbs. However, more evidence is needed to confirm whether there is an absence of social-word bias or a weakened social-word learning bias in children with ASD. In sum, the current study has contributed to the ASD and LT literature by providing further information that highlights areas of overlap and distinction in early lexical development.

## Electronic supplementary material

Below is the link to the electronic supplementary material.

Electronic supplementary material 1 (DOCX 51 kb)

## Footnotes

1 Description of the effect size statistic ${U}_{1}$ can be found in the ‘Analysis Plan’ section.

## References

[CR1] American Psychiatric Association (2013). Diagnostic and statistical manual of mental disorders.

[CR2] Bates E, Marchman V, Thal D, Fenson L, Dale P, Reznick JS (1994). Developmental and stylistic variation in the composition of early vocabulary. Journal of Child Language.

[CR3] Beckage N, Smith L, Hills T (2011). Small worlds and semantic network growth in typical and late talkers. PLoS ONE.

[CR4] Benedict H (1979). Early lexical development: Comprehension and production. Journal of Child Language.

[CR6] Bishop SL, Guthrie W, Coffing M, Lord C (2011). Convergent validity of the Mullen Scales of early learning and the differential ability scales in children with autism spectrum disorders. American Journal on Intellectual and Developmental Disabilities.

[CR5] Bishop SL, Farmer C, Thurm A (2015). Measurement of nonverbal IQ in autism spectrum disorder: Scores in young adulthood compared to early childhood. Journal of Autism and Developmental Disorders.

[CR7] Braginsky M, Yurovsky D, Marchman VA, Frank MC (2019). Consistency and variability in children’s word learning across languages. Open Mind.

[CR8] Bruckner C, Yoder P, Stone W, Saylor M (2007). Receptive vocabulary scale can be can be improved: Differential item functioning between toddlers with autism spectrum disorders and typically developing infants. Journal of Speech, Language, and Hearing Research.

[CR9] Ceponiene R, Lepistö T, Shestakova A, Vanhala R, Alku P, Näätänen R, Yaguchi K (2003). Speech-sound-selective auditory impairment in children with autism: They can perceive but do not attend. Proceedings of the National Academy of Sciences of the United States of America.

[CR10] Charman T, Drew A, Baird C, Baird G (2003). Measuring early language development in preschool children with autism spectrum disorder using the MacArthur communicative development inventory (infant form). Journal of Child Language.

[CR11] Chevallier C, Kohls G, Troiani V, Brodkin ES, Schultz RT (2012). The social motivation theory of autism introduction: Social motivation and social cognition, two competing. Trends in Cognitive Sciences.

[CR12] Clark ML, Barbaro J, Dissanayake C (2017). Continuity and change in cognition and autism severity from toddlerhood to school age. Journal of Autism and Developmental Disorders.

[CR13] Cohen J (1988). Statistical power analysis for the behavioural sciences.

[CR14] Colunga E, Sims CE (2017). Not only size matters: Early-talker and late-talker vocabularies support different word-learning biases in babies and networks. Cognitive Science.

[CR15] Conner J, Kelly-Vance L, Ryalls B, Friehe M (2014). A play and language intervention for two-year-old children: Implications for improving play skills and language. Journal of Research in Childhood Education.

[CR16] Conti-Ramsden G, Botting N, Faragher B (2001). Psycholinguistic markers for specific language impairment (SLI). Journal of Child Psychology and Psychiatry.

[CR17] Dhillon R (2010). Examining the ‘Noun Bias’: A structural approach. University of Pennsylvania Working Papers in Linguistics.

[CR18] D’odorico L, Assanelli A, Franco F, Jacob V (2007). A follow-up study on Italian late talkers: Development of language, short-term memory, phonological awareness, impulsiveness, and attention. Applied Psycholinguistics.

[CR19] Dollaghan CA (2004). Taxometric analyses of specific language impairment in 3-and 4-year-old children. Journal of Speech, Language & Hearing Research.

[CR20] Dykstra JR, Boyd BA, Watson LR, Crais ER, Baranek GT (2012). The impact of the advancing social-communication and play (ASAP) intervention on preschoolers with autism spectrum disorder. Autism.

[CR21] Eigsti IM, De Marchena AB, Schuh JM, Kelley E (2011). Language acquisition in autism spectrum disorders: A developmental review. Research in Autism Spectrum Disorders.

[CR22] Ellis Weismer S (2007). Typical talkers, late talkers, and children with specific language impairment: A language endowment spectrum. The influence of developmental perspectives on research and practice in communication disorders: A festschrift for Robin S. Chapman.

[CR23] Ellis Weismer S, Gernsbacher MA, Stronach S, Karasinski C, Eernisse ER, Venker CE, Sindberg H (2011). Lexical and grammatical skills in toddlers on the autism spectrum compared to late-talking toddlers. Journal of Autism and Developmental Disorders.

[CR24] Fenson L, Dale PS, Reznick JS, Thal D, Bates E, Hartung JP, Pethick S, Reilly JS (1993). MacArthur communicative development inventory: Users guide and technical manual.

[CR25] Field C, Allen ML, Lewis C (2016). Are children with autism spectrum disorder initially attuned to object function rather than shape for word learning?. Journal of Autism and Developmental Disorders.

[CR26] Frank MC, Braginsky M, Yurovsky D, Marchman VA (2017). Wordbank: An open repository for developmental vocabulary data. Journal of Child Language.

[CR27] Fritz CO, Morris PE, Richler JJ (2012). Effect size estimates: Current use, calculations, and interpretation. Journal of Experimental Psychology: General.

[CR28] Gernsbacher MA, Geye HM, Ellis Weismer S (2005). The role of language and communication impairments within autism. Language disorders and developmental theory.

[CR29] Goldfield BA, Reznick JS (1990). Early lexical acquisition: Rate, content, and the vocabulary spurt. Journal of Child Language.

[CR30] Hadley PA, Rispoli M, Hsu N (2016). Toddlers' verb lexicon diversity and grammatical outcomes. Language, Speech, and Hearing Services in Schools.

[CR31] Hani HB, Gonzalez-Barrero AM, Nadig AS (2013). Children's referential understanding of novel words and parent labeling behaviors: Similarities across children with and without autism spectrum disorders. Journal of Child Language.

[CR32] Hawa VV, Spanoudis G (2014). Toddlers with delayed expressive language: An overview of the characteristics, risk factors and language outcomes. Research in Developmental Disabilities.

[CR33] Horvath S, McDermott E, Reilly K, Arunachalam S (2018). Acquisition of verb meaning from syntactic distribution in preschoolers with autism spectrum disorder. Language, Speech, and Hearing Services in Schools.

[CR34] Horvath S, Rescorla L, Arunachalam S, Syrett K, Arunachalam S (2018). Semantic features of early verb vocabularies. Semantics in Language Acquisition.

[CR35] Horvath S, Rescorla L, Arunachalam S (2019). The syntactic and semantic features of two-year-olds’ verb vocabularies: A comparison of typically developing children and late talkers. Journal of Child Language.

[CR36] Ingersoll B, Schreibman L (2006). Teaching reciprocal imitation skills to young children with autism using a naturalistic behavioral approach: Effects on language, pretend play, and joint attention. Journal of Autism and Developmental Disorders.

[CR37] Irwin JR, Carter AS, Briggs-Gowan MJ (2002). The social-emotional development of “late-talking” toddlers. Journal of the American Academy of Child & Adolescent Psychiatry.

[CR38] Jones SS (2003). Late talkers show no shape bias in a novel name extension task. Developmental Science.

[CR39] Klin A (1991). Young autistic children’s listening preferences in regard to speech: A possible characterization of the symptom of social withdrawal. Journal of Autism and Developmental Disorders.

[CR40] Lazenby DC, Sideridis GD, Huntington N, Prante M, Dale PS, Curtin S (2016). Language differences at 12 months in infants who develop autism spectrum disorder. Journal of Autism and Developmental Disorders.

[CR41] Lord C, Rutter M, DiLavore PC, Risi S (1999). Autism diagnostic observation schedule.

[CR42] Lord C, Rutter M, DiLavore PC, Risi S, Gotham K, Bishop SL (2012). Autism diagnostic observation schedule, second edition (ADOS-2).

[CR43] Luyster R, Lopez K, Lord C (2007). Characterizing communicative development in children referred for autism spectrum disorders using the MacArthur-bates communicative development inventory (CDI*)*. Journal of Child Language.

[CR44] MacRoy-Higgins M, Shafer VL, Fahey KJ, Kaden ER (2016). Vocabulary of toddlers who are late talkers. Journal of Early Intervention.

[CR45] Mitchell S, Brian J, Zwaigenbaum L, Roberts W, Szatmari P, Smith I, Bryson S (2006). Early language and communication development of infants later diagnosed with autism spectrum disorder. Journal of Developmental and Behavioral Pediatrics.

[CR46] Moyle MJ, Ellis Weismer S, Evans JL, Lindstrom MJ (2007). Longitudinal relationships between lexical and grammatical development in typical and late-talking children. Journal of Speech, Language, and Hearing Research.

[CR47] Mullen EM (1995). Mullen scales of early learning.

[CR48] Naigles LR, Kelty E, Jaffery R, Fein D (2011). Abstractness and continuity in the syntactic development of young children with autism. Autism Research.

[CR49] Olswang LB, Long SH, Fletcher P (1997). Verbs in the emergence of word combinations in young children with specific expressive language impairment. International Journal of Language & Communication Disorders.

[CR50] Osterling JA, Dawson G, Munson JA (2002). Early recognition of 1-year-old infants with autism spectrum disorder versus mental retardation. Development and Psychopathology.

[CR51] Parish-Morris J (2011). Relational vocabulary in preschoolers with autistic spectrum disorder: The role of dynamic spatial concepts and social understanding.

[CR52] Paul R, Elwood TJ (1991). Maternal linguistic input to toddlers with slow expressive language development. Journal of Speech, Language, and Hearing Research.

[CR53] Payakachat N, Tilford JM, Ungar WJ (2016). National database for autism research (NDAR): Big data opportunities for health services research and health technology assessment. PharmacoEconomics.

[CR54] Pierce K, Conant D, Hazin R, Stoner R, Desmond J (2011). Preference for geometric patterns early in life as a risk factor for autism. Archives of General Psychiatry.

[CR57] Rescorla LA (1980). Overextension in early language development. Journal of Child Language.

[CR55] Rescorla L (1989). The Language Development Survey: A screening tool for delayed language in toddlers. Journal of Speech and Hearing Disorders.

[CR58] Rescorla L, Goossens M (1992). Symbolic play development in toddlers with expressive specific language impairment (SLI-E). Journal of Speech, Language, and Hearing Research.

[CR56] Rescorla L (2009). Age 17 language and reading outcomes in late-talking toddlers: Support for a dimensional perspective on language delay. Journal of Speech, Language & Hearing Research.

[CR59] Rescorla L, Safyer P (2013). Lexical composition in children with autism spectrum disorder (ASD). Journal of Child Language.

[CR60] Rescorla L, Turner HL (2015). Intentional and reactive inhibition during spoken-word stroop task performance in people with aphasia. Journal of Speech, Language, and Hearing Research.

[CR62] Rescorla L, Mirak J, Singh L (2000). Vocabulary growth in late talkers: Lexical development from 2; 0 to 3; 0. Journal of Child Language.

[CR63] Roy BC, Frank MC, DeCamp P, Miller M, Roy D (2015). Predicting the birth of a spoken word. Proceedings of the National Academy of Sciences.

[CR64] Rutter M, Le Couteur A, Lord C (2003). Autism diagnostic interview-revised.

[CR65] Schopler E, Reichler RJ, Renner BR (1988). CARS: The childhood autism rating scale.

[CR66] Shulman C, Guberman A (2007). Acquisition of verb meaning through syntactic cues: A comparison of children with autism, children with specific language impairment (SLI) and children with typical language development (TLD). Journal of Child Language.

[CR67] Swensen LD, Kelley E, Fein D, Naigles LR (2007). Processes of language acquisition in children with autism: Evidence from preferential looking. Child Development.

[CR68] Tager-Flusberg H (1992). Autistic children’s talk about psychological states: Deficits in the early acquisition of a theory of mind. Child Development.

[CR69] Tager-Flusberg H, Joseph RM (2003). Identifying neurocognitive phenotypes in autism. Philosophical Transactions of the Royal Society of London. Series B: Biological Sciences.

[CR70] Tek S, Jaffery G, Fein D, Naigles LR (2008). Do children with autism spectrum disorders show a shape bias in word learning?. Autism Research.

[CR72] Williams E, Reddy V, Costall A (2001). Taking a closer look at functional play in children with autism. Journal of Autism and Developmental Disorders.

[CR73] Ziatas K, Durkin K, Pratt C (1998). Belief term development in children with autism, Asperger syndrome, specific language impairment, and normal development: Links to theory of mind development. Journal of Child Psychology and Psychiatry.

[CR74] Snyder LS, Bates E, Bretherton I (1981). Content and context in early lexical development. Journal of Child Language.

[CR75] Happé FGE, Booth RDL (2008). The power of the positive: Revisiting weak coherence in autism spectrum disorders. Quarterly Journal of Experimental Psychology.

[CR76] Gentner, D. (1982). Why nouns are learned before verbs: Linguistic relativity versus natural partitioning. Center for the Study of Reading Technical Report; no. 257.

[CR77] Nelson K (1973). Some evidence for the cognitive primacy of categorization and its functional basis. Merrill-Palmer Quarterly of Behavior and Development.

[CR79] Huttenlocher J, Smiley P, Charney R (1983). Emergence of action categories in the child: Evidence from verb meanings. Psychological Review.

