# Supplementary material for: Identifying Areas of Overlap and Distinction in Early Lexical Profiles of Children with Autism Spectrum Disorder, Late Talkers, and Typical Talkers
Source: J Autism Dev Disord. 2020 Nov 6;51(9):3109–25. doi: 10.1007/s10803-020-04772-1 (PMC8349327; doi:10.1007/s10803-020-04772-1)
Supplement: Supplementary file 1 — Electronic supplementary material 1 (DOCX 51 kb) [file 10803_2020_4772_MOESM1_ESM.docx]

# **APPENDICES**

**Appendix A**

**Results obtained for each vocabulary bin for all syntactic classes and all semantic categories**

Table A1

Wilcoxon Rank Sum Test for each Syntactic Class

|  |  |  |  |  |  |  |  | ASD vs TT | |  | ASD vs LT | |  | TT vs LT | |
| --- | --- | --- | --- | --- | --- | --- | --- | --- | --- | --- | --- | --- | --- | --- | --- |
| Syntactic Class | Vocabulary Size | Median  ASD | Mean  ASD | Median  LT | Mean  LT | Median  TT | Mean  TT | *W* | *p* |  | *W* | *p* |  | *W* | *p* |
| Nouns | (0,25] | 0.273 | 0.318 | 0.286 | 0.321 | 0.333 | 0.351 | 24914.5 | 0.280 |  | 5707.5 | 1.132 |  | 193148.5 | 0.026 |
|  | (25,50] | 0.371 | 0.405 | 0.367 | 0.380 | 0.441 | 0.440 | 7179 | 0.543 |  | 834 | 1.142 |  | 42368 | 0.001 |
|  | (50,75] | 0.505 | 0.502 | 0.463 | 0.453 | 0.500 | 0.503 | 3005 | 1.556 |  | 413.5 | 0.524 |  | 17023 | 0.002 |
|  | (75,100] | 0.582 | 0.609 | 0.524 | 0.521 | 0.532 | 0.524 | 1103 | 0.178 |  | 67 | 0.178 |  | 3768 | 1.322 |
|  | (100,150] | 0.589 | 0.584 | 0.547 | 0.537 | 0.546 | 0.545 | 3763 | 0.602 |  | 291 | 0.602 |  | 8930.5 | 1.377 |
|  | (150,200] | 0.556 | 0.558 | 0.582 | 0.552 | 0.553 | 0.551 | 1914 | 1.989 |  | 91 | 1.989 |  | 3055 | 1.989 |
|  | (200,250] | 0.559 | 0.541 | 0.541 | 0.529 | 0.550 | 0.545 | 2093.5 | 1.839 |  | 92 | 1.282 |  | 1931 | 1.282 |
| Verbs | (0,25] | 0.048 | 0.107 | 0.000 | 0.025 | 0.000 | 0.018 | 40113 | 0.000 |  | 8092.5 | 0.000 |  | 169001 | 0.571 |
|  | (25,50] | 0.077 | 0.084 | 0.030 | 0.042 | 0.024 | 0.031 | 15090 | 0.000 |  | 1221.5 | 0.000 |  | 28761 | 0.067 |
|  | (50,75] | 0.071 | 0.085 | 0.056 | 0.059 | 0.036 | 0.046 | 4167.5 | 0.022 |  | 423.5 | 0.257 |  | 10004 | 0.022 |
|  | (75,100] | 0.106 | 0.103 | 0.052 | 0.064 | 0.052 | 0.058 | 1245 | 0.061 |  | 62 | 0.287 |  | 3286.5 | 1.128 |
|  | (100,150] | 0.062 | 0.078 | 0.069 | 0.078 | 0.071 | 0.076 | 2992 | 1.874 |  | 230 | 1.874 |  | 8516 | 1.874 |
|  | (150,200] | 0.112 | 0.107 | 0.093 | 0.096 | 0.094 | 0.098 | 2173 | 1.222 |  | 102 | 1.222 |  | 3220 | 1.402 |
|  | (200,250] | 0.117 | 0.115 | 0.121 | 0.112 | 0.102 | 0.107 | 2525.5 | 1.185 |  | 83 | 1.527 |  | 1419 | 1.185 |

*Note.* All *p*-values were first corrected using the BH method (i.e., corrections accounted for comparisons for the 3 groups of children) then corrected again using the Bonferroni method (i.e., corrections accounted for comparisons for the 2 syntactic classes).

Table A2

Wilcoxon Rank Sum Test for each Semantic Category

|  |  |  |  |  |  |  |  | ASD vs TT | |  | ASD vs LT | |  | TT vs LT | |
| --- | --- | --- | --- | --- | --- | --- | --- | --- | --- | --- | --- | --- | --- | --- | --- |
| Syntactic Class | Vocabulary Size | Median  ASD | Mean  ASD | Median  LT | Mean  LT | Median  TT | Mean  TT | *W* | *p* |  | *W* | *p* |  | *W* | *p* |
| Animals (Real or Toy) | (0,25] | 0.000 | 0.055 | 0.069 | 0.109 | 0.100 | 0.126 | 16696.0 | 0.000 |  | 4309.5 | 0.034 |  | 194996.5 | 0.034 |
|  | (25,50] | 0.074 | 0.087 | 0.068 | 0.083 | 0.103 | 0.110 | 6392.0 | 1.397 |  | 784.5 | 19.813 |  | 43113.0 | 0.002 |
|  | (50,75] | 0.113 | 0.141 | 0.088 | 0.097 | 0.107 | 0.109 | 3174.0 | 11.622 |  | 407.5 | 6.862 |  | 15285.5 | 3.159 |
|  | (75,100] | 0.112 | 0.110 | 0.094 | 0.106 | 0.105 | 0.106 | 763.0 | 21.759 |  | 43.5 | 21.759 |  | 3567.0 | 21.759 |
|  | (100,150] | 0.134 | 0.144 | 0.097 | 0.096 | 0.107 | 0.109 | 4012.5 | 1.548 |  | 323.0 | 1.548 |  | 10158.0 | 1.548 |
|  | (150,200] | 0.126 | 0.114 | 0.096 | 0.096 | 0.103 | 0.102 | 2159.0 | 11.645 |  | 104.0 | 11.645 |  | 3268.5 | 13.662 |
|  | (200,250] | 0.102 | 0.101 | 0.091 | 0.094 | 0.097 | 0.098 | 2349.0 | 14.442 |  | 94.0 | 14.442 |  | 1804.5 | 14.442 |
| Body Parts | (0,25] | 0.000 | 0.021 | 0.000 | 0.022 | 0.000 | 0.021 | 27840.5 | 14.571 |  | 5830.0 | 14.571 |  | 176117.5 | 14.571 |
|  | (25,50] | 0.036 | 0.053 | 0.043 | 0.054 | 0.038 | 0.049 | 8845.0 | 20.894 |  | 762.5 | 20.894 |  | 32739.5 | 20.894 |
|  | (50,75] | 0.055 | 0.065 | 0.056 | 0.064 | 0.065 | 0.070 | 2541.0 | 16.510 |  | 319.0 | 18.998 |  | 14298.0 | 16.510 |
|  | (75,100] | 0.047 | 0.047 | 0.069 | 0.078 | 0.074 | 0.076 | 310.0 | 2.262 |  | 16.0 | 2.262 |  | 3541.5 | 21.332 |
|  | (100,150] | 0.087 | 0.077 | 0.074 | 0.076 | 0.076 | 0.078 | 3218.5 | 17.942 |  | 250.5 | 17.942 |  | 8805.0 | 17.942 |
|  | (150,200] | 0.065 | 0.056 | 0.078 | 0.079 | 0.078 | 0.078 | 1036.5 | 1.499 |  | 57.0 | 5.520 |  | 3061.0 | 21.639 |
|  | (200,250] | 0.074 | 0.068 | 0.060 | 0.061 | 0.076 | 0.073 | 1860.0 | 9.393 |  | 92.0 | 9.393 |  | 2233.0 | 3.888 |
| Clothing | (0,25] | 0.000 | 0.011 | 0.000 | 0.014 | 0.000 | 0.017 | 27088.0 | 8.089 |  | 5995.0 | 20.202 |  | 185635.0 | 0.852 |
|  | (25,50] | 0.031 | 0.035 | 0.029 | 0.032 | 0.036 | 0.041 | 7853.0 | 14.951 |  | 796.5 | 17.923 |  | 37834.0 | 3.782 |
|  | (50,75] | 0.034 | 0.037 | 0.042 | 0.038 | 0.046 | 0.048 | 2060.5 | 3.159 |  | 308.0 | 15.950 |  | 15958.0 | 0.614 |
|  | (75,100] | 0.027 | 0.032 | 0.046 | 0.046 | 0.048 | 0.049 | 358.5 | 5.813 |  | 23.0 | 6.637 |  | 3724.5 | 16.033 |
|  | (100,150] | 0.046 | 0.043 | 0.045 | 0.042 | 0.049 | 0.050 | 2604.0 | 10.717 |  | 239.5 | 20.016 |  | 10523.0 | 1.580 |
|  | (150,200] | 0.046 | 0.053 | 0.059 | 0.055 | 0.048 | 0.049 | 1844.5 | 19.424 |  | 75.5 | 19.424 |  | 2519.0 | 14.809 |
|  | (200,250] | 0.042 | 0.048 | 0.045 | 0.046 | 0.049 | 0.049 | 1944.5 | 19.351 |  | 75.0 | 20.560 |  | 1884.0 | 19.351 |
| Connecting Words | (0,25] | 0.000 | 0.000 | 0.000 | 0.000 | 0.000 | 0.000 | 29182.5 | 18.822 |  | 6030.0 | NA |  | 174066.0 | 18.822 |
|  | (25,50] | 0.000 | 0.000 | 0.000 | 0.000 | 0.000 | 0.000 | 8680.0 | 18.355 |  | 770.0 | NA |  | 33572.0 | 18.355 |
|  | (50,75] | 0.000 | 0.000 | 0.000 | 0.001 | 0.000 | 0.000 | 2856.0 | 18.256 |  | 300.0 | 9.562 |  | 12007.0 | 0.000 |
|  | (75,100] | 0.000 | 0.000 | 0.000 | 0.001 | 0.000 | 0.000 | 696.0 | 16.925 |  | 38.0 | 16.925 |  | 3466.0 | 16.925 |
|  | (100,150] | 0.000 | 0.000 | 0.000 | 0.001 | 0.000 | 0.000 | 3003.0 | 11.412 |  | 214.5 | 9.917 |  | 8158.0 | 7.780 |
|  | (150,200] | 0.000 | 0.000 | 0.000 | 0.002 | 0.000 | 0.001 | 1630.0 | 6.993 |  | 70.0 | 6.993 |  | 2773.5 | 6.993 |
|  | (200,250] | 0.000 | 0.002 | 0.000 | 0.002 | 0.000 | 0.001 | 2355.0 | 7.830 |  | 72.0 | 16.324 |  | 1390.0 | 6.423 |
| Descriptive Words | (0,25] | 0.000 | 0.011 | 0.000 | 0.024 | 0.000 | 0.021 | 23323.0 | 0.163 |  | 5105.0 | 0.477 |  | 179749.0 | 5.606 |
|  | (25,50] | 0.034 | 0.047 | 0.034 | 0.035 | 0.029 | 0.033 | 10306.5 | 9.842 |  | 884.0 | 10.103 |  | 31885.5 | 10.455 |
|  | (50,75] | 0.037 | 0.047 | 0.048 | 0.049 | 0.037 | 0.040 | 3004.0 | 17.150 |  | 309.0 | 17.150 |  | 11042.0 | 3.414 |
|  | (75,100] | 0.046 | 0.044 | 0.052 | 0.049 | 0.044 | 0.049 | 680.5 | 19.381 |  | 35.0 | 19.381 |  | 3385.5 | 19.381 |
|  | (100,150] | 0.039 | 0.052 | 0.057 | 0.060 | 0.050 | 0.051 | 2744.5 | 10.567 |  | 173.0 | 5.631 |  | 6660.5 | 1.636 |
|  | (150,200] | 0.062 | 0.062 | 0.051 | 0.058 | 0.055 | 0.057 | 2103.5 | 15.395 |  | 100.0 | 15.395 |  | 2951.0 | 18.026 |
|  | (200,250] | 0.067 | 0.072 | 0.064 | 0.071 | 0.064 | 0.067 | 2323.0 | 21.519 |  | 78.0 | 21.519 |  | 1591.5 | 21.519 |
| Food and Drink | (0,25] | 0.083 | 0.088 | 0.000 | 0.058 | 0.000 | 0.059 | 33288.5 | 1.926 |  | 7267.0 | 0.412 |  | 190999.5 | 0.407 |
|  | (25,50] | 0.089 | 0.104 | 0.083 | 0.090 | 0.088 | 0.093 | 9423.5 | 14.390 |  | 864.0 | 14.390 |  | 34526.0 | 14.390 |
|  | (50,75] | 0.106 | 0.112 | 0.103 | 0.106 | 0.107 | 0.110 | 2664.0 | 21.569 |  | 328.0 | 21.569 |  | 13986.0 | 21.569 |
|  | (75,100] | 0.236 | 0.241 | 0.114 | 0.125 | 0.114 | 0.114 | 1400.5 | 0.059 |  | 78.0 | 0.121 |  | 3191.5 | 9.603 |
|  | (100,150] | 0.140 | 0.142 | 0.133 | 0.131 | 0.119 | 0.122 | 3992.5 | 2.948 |  | 257.5 | 13.253 |  | 7128.5 | 2.948 |
|  | (150,200] | 0.138 | 0.149 | 0.117 | 0.130 | 0.125 | 0.126 | 2329.0 | 7.182 |  | 102.0 | 13.442 |  | 3025.5 | 20.978 |
|  | (200,250] | 0.123 | 0.129 | 0.154 | 0.135 | 0.127 | 0.127 | 2241.5 | 19.604 |  | 74.0 | 19.604 |  | 1370.0 | 19.604 |
| Furniture and Rooms | (0,25] | 0.000 | 0.003 | 0.000 | 0.002 | 0.000 | 0.004 | 28660.5 | 13.557 |  | 6184.5 | 9.388 |  | 181868.5 | 0.220 |
|  | (25,50] | 0.000 | 0.015 | 0.000 | 0.011 | 0.000 | 0.011 | 9595.0 | 13.459 |  | 819.0 | 13.459 |  | 32126.0 | 13.459 |
|  | (50,75] | 0.023 | 0.026 | 0.016 | 0.017 | 0.015 | 0.018 | 3527.5 | 6.364 |  | 408.5 | 6.364 |  | 13101.5 | 21.283 |
|  | (75,100] | 0.039 | 0.033 | 0.023 | 0.023 | 0.022 | 0.024 | 947.5 | 8.426 |  | 58.0 | 8.426 |  | 3445.5 | 17.802 |
|  | (100,150] | 0.035 | 0.036 | 0.027 | 0.033 | 0.029 | 0.031 | 3543.5 | 15.236 |  | 267.0 | 15.236 |  | 8520.0 | 20.661 |
|  | (150,200] | 0.040 | 0.041 | 0.043 | 0.043 | 0.037 | 0.038 | 1987.0 | 18.641 |  | 82.0 | 19.802 |  | 2586.0 | 18.641 |
|  | (200,250] | 0.046 | 0.046 | 0.036 | 0.034 | 0.040 | 0.042 | 2324.0 | 12.344 |  | 95.0 | 11.152 |  | 2021.0 | 11.152 |
| Games and Routines | (0,25] | 0.125 | 0.153 | 0.132 | 0.141 | 0.158 | 0.156 | 25809.5 | 6.024 |  | 5914.5 | 18.364 |  | 190490.5 | 0.892 |
|  | (25,50] | 0.126 | 0.121 | 0.146 | 0.156 | 0.136 | 0.139 | 7362.0 | 5.264 |  | 516.5 | 0.794 |  | 27785.5 | 0.794 |
|  | (50,75] | 0.095 | 0.080 | 0.113 | 0.116 | 0.111 | 0.112 | 1836.0 | 1.105 |  | 199.5 | 1.105 |  | 12407.5 | 10.903 |
|  | (75,100] | 0.059 | 0.061 | 0.093 | 0.090 | 0.094 | 0.095 | 321.5 | 3.928 |  | 21.5 | 5.381 |  | 3614.0 | 20.019 |
|  | (100,150] | 0.066 | 0.061 | 0.077 | 0.079 | 0.078 | 0.080 | 2105.0 | 2.036 |  | 151.0 | 2.036 |  | 8547.0 | 21.212 |
|  | (150,200] | 0.067 | 0.068 | 0.067 | 0.067 | 0.071 | 0.071 | 1588.0 | 13.932 |  | 95.0 | 13.932 |  | 3482.5 | 13.932 |
|  | (200,250] | 0.054 | 0.050 | 0.066 | 0.063 | 0.059 | 0.061 | 1399.0 | 1.942 |  | 42.0 | 1.942 |  | 1506.5 | 12.720 |
| Helping Verbs | (0,25] | 0.000 | 0.005 | 0.000 | 0.002 | 0.000 | 0.002 | 29705.5 | 16.551 |  | 6083.0 | 17.133 |  | 172455.0 | 16.551 |
|  | (25,50] | 0.000 | 0.006 | 0.000 | 0.006 | 0.000 | 0.003 | 9811.5 | 5.263 |  | 832.5 | 8.889 |  | 32053.0 | 8.645 |
|  | (50,75] | 0.000 | 0.005 | 0.000 | 0.008 | 0.000 | 0.004 | 3020.5 | 19.894 |  | 324.0 | 19.894 |  | 12339.0 | 19.293 |
|  | (75,100] | 0.005 | 0.005 | 0.012 | 0.014 | 0.000 | 0.005 | 783.0 | 14.876 |  | 18.5 | 3.282 |  | 1858.5 | 0.001 |
|  | (100,150] | 0.000 | 0.004 | 0.007 | 0.006 | 0.000 | 0.005 | 3015.0 | 18.631 |  | 194.0 | 11.113 |  | 7609.5 | 11.113 |
|  | (150,200] | 0.005 | 0.004 | 0.000 | 0.007 | 0.005 | 0.006 | 1791.0 | 21.806 |  | 89.0 | 21.806 |  | 3185.5 | 21.806 |
|  | (200,250] | 0.005 | 0.008 | 0.010 | 0.010 | 0.004 | 0.007 | 2134.5 | 21.680 |  | 37.0 | 1.014 |  | 1043.0 | 1.014 |
| Outside things | (0,25] | 0.000 | 0.009 | 0.000 | 0.004 | 0.000 | 0.007 | 29089.0 | 20.452 |  | 6179.5 | 18.133 |  | 179191.0 | 7.816 |
|  | (25,50] | 0.024 | 0.027 | 0.020 | 0.022 | 0.000 | 0.019 | 10095.0 | 5.841 |  | 835.5 | 11.907 |  | 30614.0 | 5.841 |
|  | (50,75] | 0.033 | 0.032 | 0.019 | 0.025 | 0.027 | 0.029 | 3036.0 | 16.025 |  | 378.5 | 14.167 |  | 14305.5 | 14.167 |
|  | (75,100] | 0.036 | 0.036 | 0.040 | 0.036 | 0.035 | 0.037 | 710.0 | 21.873 |  | 41.0 | 21.873 |  | 3513.5 | 21.873 |
|  | (100,150] | 0.053 | 0.043 | 0.045 | 0.044 | 0.043 | 0.043 | 3139.5 | 21.007 |  | 237.0 | 21.007 |  | 8351.0 | 21.007 |
|  | (150,200] | 0.053 | 0.053 | 0.032 | 0.040 | 0.045 | 0.044 | 2267.5 | 4.394 |  | 117.0 | 4.394 |  | 3613.5 | 4.394 |
|  | (200,250] | 0.056 | 0.058 | 0.043 | 0.043 | 0.049 | 0.048 | 2920.0 | 0.876 |  | 118.0 | 0.876 |  | 1909.5 | 9.339 |
| People | (0,25] | 0.143 | 0.160 | 0.185 | 0.203 | 0.167 | 0.180 | 26072.0 | 4.827 |  | 5032.0 | 2.446 |  | 157084.5 | 0.805 |
|  | (25,50] | 0.093 | 0.093 | 0.111 | 0.115 | 0.108 | 0.112 | 6786.0 | 3.044 |  | 561.5 | 3.044 |  | 31664.5 | 9.378 |
|  | (50,75] | 0.056 | 0.068 | 0.093 | 0.085 | 0.082 | 0.083 | 1933.5 | 3.035 |  | 226.5 | 3.035 |  | 11985.5 | 6.246 |
|  | (75,100] | 0.064 | 0.058 | 0.062 | 0.063 | 0.066 | 0.067 | 605.0 | 17.295 |  | 36.0 | 17.295 |  | 3948.0 | 17.295 |
|  | (100,150] | 0.048 | 0.045 | 0.054 | 0.055 | 0.056 | 0.057 | 1996.5 | 1.879 |  | 172.0 | 5.401 |  | 9211.5 | 10.255 |
|  | (150,200] | 0.041 | 0.038 | 0.039 | 0.046 | 0.046 | 0.046 | 1175.0 | 4.131 |  | 67.5 | 10.475 |  | 3364.0 | 10.475 |
|  | (200,250] | 0.036 | 0.038 | 0.042 | 0.041 | 0.042 | 0.044 | 1543.5 | 5.446 |  | 56.0 | 8.635 |  | 1759.0 | 16.955 |
| Places to Go | (0,25] | 0.000 | 0.003 | 0.000 | 0.002 | 0.000 | 0.003 | 28445.0 | 14.011 |  | 6053.0 | 19.054 |  | 179191.5 | 1.862 |
|  | (25,50] | 0.000 | 0.007 | 0.000 | 0.007 | 0.000 | 0.007 | 9059.5 | 20.514 |  | 802.5 | 20.514 |  | 33640.5 | 20.514 |
|  | (50,75] | 0.007 | 0.009 | 0.000 | 0.011 | 0.013 | 0.011 | 2870.5 | 21.923 |  | 337.0 | 21.923 |  | 13372.5 | 21.923 |
|  | (75,100] | 0.021 | 0.021 | 0.012 | 0.016 | 0.012 | 0.015 | 960.5 | 10.913 |  | 53.0 | 10.913 |  | 3465.0 | 18.483 |
|  | (100,150] | 0.009 | 0.016 | 0.022 | 0.022 | 0.018 | 0.018 | 2719.5 | 9.893 |  | 177.0 | 6.615 |  | 7155.5 | 6.286 |
|  | (150,200] | 0.022 | 0.024 | 0.017 | 0.020 | 0.021 | 0.021 | 2002.5 | 11.744 |  | 101.5 | 11.744 |  | 3441.5 | 11.744 |
|  | (200,250] | 0.024 | 0.023 | 0.027 | 0.030 | 0.023 | 0.024 | 2129.0 | 21.974 |  | 44.0 | 2.478 |  | 926.5 | 0.795 |
| Prepositions and Locations | (0,25] | 0.000 | 0.028 | 0.000 | 0.017 | 0.000 | 0.014 | 33006.0 | 1.437 |  | 6777.0 | 1.437 |  | 174366.5 | 20.242 |
|  | (25,50] | 0.021 | 0.025 | 0.020 | 0.027 | 0.000 | 0.021 | 9563.5 | 13.274 |  | 778.0 | 20.754 |  | 30600.5 | 10.954 |
|  | (50,75] | 0.031 | 0.026 | 0.027 | 0.025 | 0.018 | 0.023 | 3096.5 | 20.398 |  | 336.0 | 20.398 |  | 12382.5 | 20.398 |
|  | (75,100] | 0.027 | 0.024 | 0.013 | 0.019 | 0.023 | 0.024 | 718.0 | 21.532 |  | 45.5 | 21.532 |  | 4097.0 | 16.797 |
|  | (100,150] | 0.017 | 0.017 | 0.016 | 0.021 | 0.023 | 0.023 | 2446.0 | 6.384 |  | 220.5 | 16.869 |  | 9774.0 | 6.384 |
|  | (150,200] | 0.025 | 0.020 | 0.025 | 0.025 | 0.022 | 0.022 | 1654.5 | 14.823 |  | 72.0 | 14.823 |  | 2635.5 | 14.823 |
|  | (200,250] | 0.015 | 0.020 | 0.018 | 0.021 | 0.021 | 0.021 | 1831.0 | 15.721 |  | 63.5 | 15.721 |  | 1737.0 | 18.214 |
| Pronouns | (0,25] | 0.000 | 0.011 | 0.000 | 0.018 | 0.000 | 0.018 | 27418.0 | 12.588 |  | 5701.0 | 12.588 |  | 175001.0 | 17.999 |
|  | (25,50] | 0.022 | 0.027 | 0.026 | 0.028 | 0.000 | 0.023 | 9243.5 | 13.351 |  | 673.0 | 12.616 |  | 27124.5 | 0.204 |
|  | (50,75] | 0.025 | 0.034 | 0.029 | 0.033 | 0.017 | 0.021 | 3310.0 | 11.623 |  | 311.5 | 16.884 |  | 9884.5 | 0.142 |
|  | (75,100] | 0.000 | 0.003 | 0.013 | 0.019 | 0.017 | 0.020 | 216.5 | 0.527 |  | 5.0 | 0.475 |  | 3477.0 | 18.935 |
|  | (100,150] | 0.016 | 0.017 | 0.022 | 0.020 | 0.017 | 0.017 | 2574.5 | 6.499 |  | 163.0 | 4.169 |  | 7277.5 | 4.169 |
|  | (150,200] | 0.014 | 0.012 | 0.016 | 0.018 | 0.016 | 0.016 | 1391.5 | 14.893 |  | 71.0 | 16.384 |  | 3081.5 | 20.818 |
|  | (200,250] | 0.008 | 0.012 | 0.024 | 0.021 | 0.015 | 0.016 | 1519.0 | 1.974 |  | 37.5 | 1.974 |  | 1168.0 | 1.974 |
| Quantifiers and Articles | (0,25] | 0.000 | 0.010 | 0.000 | 0.006 | 0.000 | 0.005 | 30998.0 | 4.271 |  | 6423.5 | 4.271 |  | 174830.5 | 16.882 |
|  | (25,50] | 0.022 | 0.019 | 0.000 | 0.012 | 0.000 | 0.008 | 11283.5 | 0.323 |  | 925.5 | 2.780 |  | 29847.0 | 1.664 |
|  | (50,75] | 0.017 | 0.018 | 0.000 | 0.011 | 0.000 | 0.009 | 3652.0 | 5.037 |  | 396.5 | 8.252 |  | 12425.0 | 10.239 |
|  | (75,100] | 0.005 | 0.006 | 0.005 | 0.008 | 0.011 | 0.009 | 545.0 | 13.428 |  | 36.0 | 16.979 |  | 4016.0 | 13.428 |
|  | (100,150] | 0.007 | 0.008 | 0.008 | 0.009 | 0.008 | 0.007 | 2611.5 | 9.762 |  | 186.5 | 9.762 |  | 7943.0 | 9.762 |
|  | (150,200] | 0.011 | 0.010 | 0.006 | 0.011 | 0.006 | 0.008 | 2325.0 | 7.208 |  | 106.5 | 9.599 |  | 2826.0 | 13.311 |
|  | (200,250] | 0.006 | 0.011 | 0.009 | 0.011 | 0.008 | 0.008 | 1952.0 | 13.230 |  | 62.0 | 13.230 |  | 1483.5 | 13.230 |
| Question Words | (0,25] | 0.000 | 0.002 | 0.000 | 0.002 | 0.000 | 0.005 | 28067.0 | 9.276 |  | 5964.0 | 15.442 |  | 178928.0 | 4.271 |
|  | (25,50] | 0.000 | 0.006 | 0.000 | 0.006 | 0.000 | 0.005 | 9096.5 | 16.652 |  | 793.5 | 16.652 |  | 33018.0 | 16.652 |
|  | (50,75] | 0.007 | 0.017 | 0.000 | 0.004 | 0.000 | 0.004 | 3745.0 | 0.711 |  | 424.0 | 1.799 |  | 12821.5 | 14.778 |
|  | (75,100] | 0.000 | 0.003 | 0.000 | 0.009 | 0.000 | 0.005 | 655.0 | 16.126 |  | 32.5 | 16.126 |  | 3105.0 | 15.299 |
|  | (100,150] | 0.007 | 0.008 | 0.000 | 0.005 | 0.000 | 0.003 | 3913.0 | 1.812 |  | 266.0 | 9.760 |  | 7225.5 | 1.812 |
|  | (150,200] | 0.003 | 0.004 | 0.005 | 0.007 | 0.000 | 0.004 | 1907.0 | 15.516 |  | 75.0 | 15.516 |  | 2462.5 | 8.590 |
|  | (200,250] | 0.000 | 0.005 | 0.005 | 0.007 | 0.002 | 0.004 | 2197.5 | 18.176 |  | 60.0 | 11.385 |  | 1138.5 | 3.673 |
| Small Household Items | (0,25] | 0.000 | 0.013 | 0.000 | 0.024 | 0.000 | 0.037 | 22106.5 | 0.033 |  | 5697.0 | 7.593 |  | 204735.5 | 0.000 |
|  | (25,50] | 0.000 | 0.016 | 0.021 | 0.025 | 0.038 | 0.044 | 4594.5 | 0.009 |  | 649.0 | 5.581 |  | 44014.5 | 0.000 |
|  | (50,75] | 0.038 | 0.047 | 0.046 | 0.047 | 0.055 | 0.059 | 2341.0 | 9.144 |  | 322.0 | 19.852 |  | 15571.5 | 1.642 |
|  | (75,100] | 0.057 | 0.061 | 0.052 | 0.053 | 0.063 | 0.068 | 603.5 | 19.859 |  | 42.0 | 19.965 |  | 4504.5 | 3.031 |
|  | (100,150] | 0.037 | 0.054 | 0.070 | 0.072 | 0.074 | 0.075 | 1864.0 | 0.933 |  | 145.0 | 1.486 |  | 9183.5 | 10.700 |
|  | (150,200] | 0.073 | 0.068 | 0.077 | 0.078 | 0.081 | 0.082 | 1248.5 | 6.651 |  | 71.0 | 13.274 |  | 3279.5 | 13.274 |
|  | (200,250] | 0.083 | 0.080 | 0.068 | 0.071 | 0.088 | 0.086 | 1936.0 | 12.521 |  | 92.0 | 12.521 |  | 2295.5 | 2.361 |
| Sound Effects and Animal Sounds | (0,25] | 0.143 | 0.183 | 0.222 | 0.235 | 0.200 | 0.220 | 24812.0 | 2.821 |  | 5021.0 | 2.821 |  | 165917.5 | 5.142 |
|  | (25,50] | 0.115 | 0.129 | 0.152 | 0.163 | 0.154 | 0.157 | 6485.5 | 2.839 |  | 577.0 | 2.839 |  | 33245.5 | 20.104 |
|  | (50,75] | 0.067 | 0.071 | 0.121 | 0.116 | 0.115 | 0.113 | 1402.5 | 0.104 |  | 149.0 | 0.104 |  | 12346.0 | 10.128 |
|  | (75,100] | 0.017 | 0.026 | 0.091 | 0.088 | 0.092 | 0.090 | 57.0 | 0.103 |  | 2.5 | 0.135 |  | 3811.5 | 13.103 |
|  | (100,150] | 0.061 | 0.061 | 0.061 | 0.060 | 0.071 | 0.070 | 2552.0 | 9.128 |  | 247.0 | 17.097 |  | 10661.0 | 1.027 |
|  | (150,200] | 0.041 | 0.037 | 0.053 | 0.048 | 0.055 | 0.053 | 734.0 | 0.095 |  | 51.5 | 3.215 |  | 3593.0 | 4.766 |
|  | (200,250] | 0.042 | 0.039 | 0.042 | 0.038 | 0.045 | 0.043 | 1994.5 | 15.880 |  | 84.0 | 15.880 |  | 1915.5 | 15.880 |
| Toys | (0,25] | 0.000 | 0.076 | 0.043 | 0.072 | 0.056 | 0.074 | 26935.0 | 11.772 |  | 5772.0 | 13.724 |  | 180091.5 | 11.772 |
|  | (25,50] | 0.049 | 0.054 | 0.044 | 0.047 | 0.065 | 0.067 | 6762.0 | 2.911 |  | 910.0 | 4.692 |  | 44596.5 | 0.000 |
|  | (50,75] | 0.050 | 0.043 | 0.046 | 0.049 | 0.058 | 0.059 | 1997.0 | 2.387 |  | 322.0 | 19.852 |  | 17341.0 | 0.007 |
|  | (75,100] | 0.056 | 0.052 | 0.052 | 0.051 | 0.050 | 0.051 | 741.0 | 21.320 |  | 41.0 | 21.320 |  | 3501.5 | 21.320 |
|  | (100,150] | 0.045 | 0.049 | 0.040 | 0.039 | 0.045 | 0.046 | 3160.5 | 19.931 |  | 286.0 | 8.032 |  | 10619.5 | 1.172 |
|  | (150,200] | 0.035 | 0.038 | 0.029 | 0.035 | 0.040 | 0.041 | 1486.0 | 7.783 |  | 104.5 | 7.783 |  | 4075.5 | 1.281 |
|  | (200,250] | 0.037 | 0.035 | 0.052 | 0.050 | 0.038 | 0.039 | 1803.0 | 7.363 |  | 25.0 | 0.158 |  | 710.0 | 0.079 |
| Vehicles (Real or Toy) | (0,25] | 0.000 | 0.050 | 0.000 | 0.020 | 0.000 | 0.014 | 31337.5 | 9.955 |  | 6316.5 | 9.955 |  | 169542.5 | 9.955 |
|  | (25,50] | 0.034 | 0.041 | 0.034 | 0.039 | 0.022 | 0.025 | 10798.5 | 1.774 |  | 781.5 | 20.255 |  | 25856.0 | 0.035 |
|  | (50,75] | 0.027 | 0.030 | 0.032 | 0.035 | 0.029 | 0.031 | 2921.0 | 20.093 |  | 305.5 | 20.093 |  | 11907.5 | 16.551 |
|  | (75,100] | 0.034 | 0.033 | 0.036 | 0.039 | 0.034 | 0.035 | 670.5 | 18.545 |  | 34.0 | 18.545 |  | 3214.0 | 18.545 |
|  | (100,150] | 0.039 | 0.039 | 0.047 | 0.048 | 0.034 | 0.035 | 3238.5 | 17.267 |  | 184.5 | 8.813 |  | 5333.5 | 0.010 |
|  | (150,200] | 0.038 | 0.039 | 0.037 | 0.036 | 0.032 | 0.034 | 2189.0 | 15.636 |  | 92.0 | 16.370 |  | 2758.5 | 16.370 |
|  | (200,250] | 0.033 | 0.034 | 0.040 | 0.039 | 0.032 | 0.032 | 2317.0 | 12.654 |  | 61.0 | 12.654 |  | 1111.0 | 3.888 |
| Words About Time | (0,25] | 0.000 | 0.000 | 0.000 | 0.000 | 0.000 | 0.000 | 29025.0 | 15.151 |  | 6007.5 | 15.151 |  | 174356.0 | 15.151 |
|  | (25,50] | 0.000 | 0.001 | 0.000 | 0.001 | 0.000 | 0.001 | 8845.5 | 22.000 |  | 769.5 | 22.000 |  | 32885.0 | 22.000 |
|  | (50,75] | 0.000 | 0.005 | 0.000 | 0.003 | 0.000 | 0.002 | 3293.5 | 3.422 |  | 352.5 | 13.250 |  | 12070.5 | 3.422 |
|  | (75,100] | 0.000 | 0.003 | 0.000 | 0.005 | 0.000 | 0.002 | 758.0 | 16.313 |  | 33.0 | 16.313 |  | 2765.0 | 0.916 |
|  | (100,150] | 0.000 | 0.006 | 0.000 | 0.003 | 0.000 | 0.003 | 2980.5 | 16.802 |  | 215.0 | 16.802 |  | 8269.0 | 16.802 |
|  | (150,200] | 0.005 | 0.004 | 0.000 | 0.004 | 0.000 | 0.003 | 2100.0 | 10.587 |  | 103.5 | 10.587 |  | 3149.5 | 17.629 |
|  | (200,250] | 0.004 | 0.006 | 0.000 | 0.003 | 0.004 | 0.005 | 2108.0 | 20.948 |  | 87.5 | 18.311 |  | 2004.0 | 16.304 |

*Note.* All *p*-values were first corrected using the BH method (i.e., corrections accounted for comparisons for the 3 groups of children) then corrected again using the Bonferroni method (i.e., corrections accounted for comparisons for the 22 CDI semantic classes). Action Words not displayed as shown as Verbs in the syntactic classes comparison.

**Appendix B**

**Results obtained for each syntactic class, classified using Bates et al.’s approach (1994)**

Table B

Wilcoxon Rank Sum Test for each Syntactic Class

|  |  |  |  |  |  |  |  | ASD vs TT | |  | ASD vs LT | |  | TT vs LT | |
| --- | --- | --- | --- | --- | --- | --- | --- | --- | --- | --- | --- | --- | --- | --- | --- |
| Syntactic Class | Vocabulary Size | Median  ASD | Mean  ASD | Median  LT | Mean  LT | Median  TT | Mean  TT | *W* | *p* |  | *W* | *p* |  | *W* | *p* |
| Nouns | (0,25] | 0.769 | 0.660 | 0.854 | 0.778 | 0.875 | 0.813 | 22526.0 | 0.058 |  | 4909.0 | 0.160 |  | 179629.0 | 1.130 |
|  | (25,50] | 0.650 | 0.654 | 0.727 | 0.714 | 0.800 | 0.781 | 4573.5 | 0.001 |  | 585.0 | 0.299 |  | 42366.5 | 0.001 |
|  | (50,75] | 0.723 | 0.688 | 0.697 | 0.714 | 0.786 | 0.777 | 2151.5 | 0.627 |  | 329.0 | 2.980 |  | 17375.0 | 0.001 |
|  | (75,100] | 0.769 | 0.769 | 0.750 | 0.756 | 0.768 | 0.759 | 728.5 | 2.815 |  | 43.5 | 2.815 |  | 3666.5 | 2.815 |
|  | (100,150] | 0.813 | 0.763 | 0.743 | 0.736 | 0.756 | 0.753 | 3636.0 | 1.038 |  | 289.5 | 1.038 |  | 9394.5 | 1.038 |
|  | (150,200] | 0.714 | 0.723 | 0.714 | 0.719 | 0.733 | 0.730 | 1606.0 | 2.581 |  | 81.0 | 2.581 |  | 3243.0 | 2.581 |
|  | (200,250] | 0.728 | 0.695 | 0.696 | 0.687 | 0.714 | 0.709 | 2088.5 | 2.723 |  | 88.0 | 2.544 |  | 1914.5 | 2.544 |
| Predicates | (0,25] | 0.100 | 0.214 | 0.000 | 0.120 | 0.000 | 0.094 | 35810.5 | 0.031 |  | 7231.5 | 0.066 |  | 171396.0 | 2.003 |
|  | (25,50] | 0.231 | 0.218 | 0.130 | 0.141 | 0.095 | 0.117 | 13690.0 | 0.000 |  | 1119.0 | 0.007 |  | 26466.0 | 0.007 |
|  | (50,75] | 0.155 | 0.182 | 0.177 | 0.170 | 0.122 | 0.133 | 3550.0 | 0.718 |  | 344.0 | 2.476 |  | 9478.5 | 0.006 |
|  | (75,100] | 0.175 | 0.186 | 0.162 | 0.165 | 0.151 | 0.157 | 941.0 | 1.684 |  | 48.0 | 1.684 |  | 3268.5 | 1.684 |
|  | (100,150] | 0.138 | 0.172 | 0.180 | 0.189 | 0.172 | 0.176 | 2784.5 | 1.593 |  | 191.5 | 1.558 |  | 7777.5 | 1.558 |
|  | (150,200] | 0.222 | 0.218 | 0.208 | 0.200 | 0.203 | 0.204 | 2175.5 | 1.140 |  | 114.0 | 1.140 |  | 3162.5 | 2.402 |
|  | (200,250] | 0.241 | 0.242 | 0.239 | 0.236 | 0.220 | 0.226 | 2514.5 | 1.696 |  | 88.0 | 1.696 |  | 1435.0 | 1.696 |
| Closed class | (0,25] | 0.000 | 0.126 | 0.000 | 0.102 | 0.000 | 0.093 | 31531.0 | 1.393 |  | 6511.5 | 1.393 |  | 174445.0 | 2.786 |
|  | (25,50] | 0.099 | 0.128 | 0.143 | 0.146 | 0.087 | 0.102 | 10195.0 | 0.832 |  | 728.5 | 2.143 |  | 26018.0 | 0.010 |
|  | (50,75] | 0.108 | 0.130 | 0.119 | 0.116 | 0.082 | 0.090 | 3241.0 | 1.959 |  | 302.0 | 1.959 |  | 10183.0 | 0.055 |
|  | (75,100] | 0.054 | 0.045 | 0.075 | 0.079 | 0.077 | 0.084 | 359.5 | 0.801 |  | 25.0 | 1.176 |  | 3758.5 | 2.026 |
|  | (100,150] | 0.052 | 0.065 | 0.068 | 0.076 | 0.067 | 0.071 | 2493.5 | 1.029 |  | 180.0 | 1.029 |  | 8255.0 | 2.100 |
|  | (150,200] | 0.058 | 0.059 | 0.074 | 0.081 | 0.063 | 0.066 | 1602.5 | 1.692 |  | 71.5 | 1.692 |  | 2782.5 | 1.692 |
|  | (200,250] | 0.041 | 0.063 | 0.071 | 0.077 | 0.061 | 0.065 | 1640.5 | 0.443 |  | 48.5 | 0.443 |  | 1169.0 | 0.443 |

*Note.*All *p*-values were first corrected using the BH method (i.e., corrections accounted for comparisons for the 3 groups of children) then corrected again using the Bonferroni method (i.e., corrections accounted for comparisons for the 3 syntactic classes).

**Appendix C**

**Results obtained to Identify Words which are Potentially Related to Differences in Development**

Table C

*Top-10 Words that Older and Younger Children Differed the Most in Production per Vocabulary Size*

| Group | (0,25] | (25,50] | (50,75] | (75,100] | (100,150] | (150,200] | (200,250] |
| --- | --- | --- | --- | --- | --- | --- | --- |
| Older | mommy | yes | bee | cereal | ice-cream | jacket | lion |
|  | daddy | choo-choo | potty | candy | helicopter | orange (description) | red |
|  | go | please | help | horse | candy | red | jump |
|  | uhoh | go | go-potty | TV | help | shorts | playdough |
|  | ball | ouch | mine | bed | hurt | swing (action) | snow |
|  | ouch | shush | go | drink (food) | TV | blue | black |
|  | car | car | eat | go | pants | plate | chicken (animal) |
|  | moo | hello | please | truck | broken | clean (action) | penguin |
|  | mine | blue | candy | gum | motorcycle | open | stop |
|  | shush | mine | me | hug | bus | ice-cream | broken |
| Younger | bottle | bottle | bear | bottle | yum | doll | owiebooboo |
|  | hi | book | balloon | cracker | cheese | vroom | pig |
|  | kitty | dog | cracker | button | bear | bunny | toast |
|  | duck | kitty | juice | baa | doll | broom | bib |
|  | balloon | cracker | cheese | out | block | nice | drink (action) |
|  | baby | bird | bottle | bird | bellybutton | toy | call (phone) |
|  | bird | balloon | bath | peas | bath | walk | owl |
|  | yum | cat | airplane | yum | toe | ouch | belt |
|  | that | woof | yum | that | spoon | mine | buttocks/bottom |
|  | book | duck | teddy bear | keys | hat | buttocks/bottom | bellybutton |

Note. The above table shows the top-ten words that each group produced more of compared to their size matched equivalent younger or older group.
